# Ustekinumab for type 1 diabetes in adolescents: a multicenter, double-blind, randomized phase 2 trial

**DOI:** 10.1038/s41591-024-03115-2

**Published:** 2024-07-30

**Authors:** Danijela Tatovic, Ashish Marwaha, Peter Taylor, Stephanie J. Hanna, Kym Carter, W. Y. Cheung, Steve Luzio, Gareth Dunseath, Hayley A. Hutchings, Gail Holland, Steve Hiles, Greg Fegan, Evangelia Williams, Jennie H. M. Yang, Clara Domingo-Vila, Emily Pollock, Muntaha Wadud, Kirsten Ward-Hartstonge, Susie Marques-Jones, Jane Bowen-Morris, Rachel Stenson, Megan K. Levings, John W. Gregory, Timothy I. M. Tree, Colin Dayan, USTEKID Study Group, Evelien Gevers, Shankar Kanumakala, Sunil Nair, Chris Gardner, Michal Ajzensztejn, Christina Wei, Chris Mouditis, Fiona Campbell, James Greening, Emma Webb, Mimi Chen, Rakesh Amin, Billi White, Ambika Shetty, Chris Bidder, Nicholas Conway, Amalia Mayo, Eleni Christakou, Kamila Sychowska, Yasaman Shahrabi, Maximilian Robinson, Simi Ahmed, Jan Dutz, Laura Cook

**Affiliations:** 1 Division of Infection and Immunity, Cardiff University School of Medicine, Cardiff, UK; 2 University of Calgary, Calgary, Alberta Canada; 3 Diabetes Research Unit Cymru, Institute for Life Sciences, Swansea University, Swansea, UK; 4 Swansea Trials Unit, Swansea University Medical School, Swansea, UK; 5 Department of Immunobiology, School of Immunology & Microbial Sciences, King’s College London, Guy’s Hospital, London, UK; 6 BC Children’s Hospital Research Institute, Vancouver, British Columbia Canada; 7 Department of Surgery, University of British Columbia, Vancouver, British Columbia Canada; 8 Patient and Public Representative, Ammanford, UK; 9 Division of Population Medicine, Cardiff University School of Medicine, Cardiff, UK; 10 Royal London Hospital, London, UK; 11 Royal Alexandra Children’s Hospital, Brighton, UK; 12 Countess of Chester Hospital, Chester, UK; 13 East Lancashire Hospitals NHS Trust, Burnley, UK; 14 The Evelina London Children’s Hospital, London, UK; 15 Royal Devon and Exeter Hospital, Exeter, UK; 16 St James’ Hospital, Leeds, UK; 17 Leicester Royal Infirmary, Leicester, UK; 18 Norfolk and Norwich University Hospitals, Norwich, UK; 19 St George’s University NHS Trust, London, UK; 20 University College London, London, UK; 21 Noah’s Ark Children’s Hospital, Cardiff, UK; 22 Swansea Bay University Health Board, Swansea, UK; 23 Ninewells Hospital, Dundee, UK; 24 Royal Aberdeen Children’s Hospital, Aberdeen, UK; 25 Kings College London, London, UK; 26 Breakthrough T1D (formerly Juvenile Diabetes Research Foundation International), New York, NY USA; 27 BC Children’s Hospital Research Institute, Vancouver, University of British Columbia, Vancouver, British Columbia Canada

**Keywords:** Diabetes, Autoimmunity

## Abstract

Immunotherapy targeting the autoimmune process in type 1 diabetes (T1D) can delay the loss of β-cells but needs to have minimal adverse effects to be an adjunct to insulin in the management of T1D. Ustekinumab binds to the shared p40 subunit of interleukin (IL)-12 and IL-23, targeting development of T helper 1 cells and T helper 17 cells (T_H_1 and T_H_17 cells) implicated in the pathogenesis of T1D. We conducted a double-blind, randomized controlled trial of ustekinumab in 72 adolescents aged 12–18 years with recent-onset T1D. Treatment was well tolerated with no increase in adverse events. At 12 months, β-cell function, measured by stimulated C-peptide, was 49% higher in the intervention group (*P* = 0.02), meeting the prespecified primary outcome. Preservation of C-peptide correlated with the reduction of T helper cells co-secreting IL-17A and interferon-γ (T_H_17.1 cells, *P* = 0.04) and, in particular, with the reduction in a subset of T_H_17.1 cells co-expressing IL-2 and granulocyte–macrophage colony-stimulating factor (IL-2^+^ GM-CSF^+^ T_H_17.1 cells, *P* = 0.04). A significant fall in β-cell-targeted (proinsulin-specific) IL-17A-secreting T cells was also seen (*P* = 0.0003). Although exploratory, our data suggest a role for an activated subset of T_H_17.1 cells in T1D that can be targeted with minimal adverse effects to reduce C-peptide loss, which requires confirmation in a larger study. (International Standard Randomised Controlled Trial Number Registry: ISRCTN 14274380).

## Abstract (web summary)

A phase 2 randomized controlled trial of ustekinumab in 72 adolescents with recent-onset type 1 diabetes showed that treatment was well tolerated and β-cell function was 49% higher in the intervention group compared to the placebo arm after 12 months.

## Main

The autoimmune, T cell-mediated destruction of insulin-producing β-cells causes T1D. In contrast to other autoimmune conditions, where immunomodulatory therapy has been established, the mainstay of T1D treatment for >100 years has been insulin replacement despite a suboptimal effect on glycemic control in many patients, especially in younger individuals^1,2^. It has been widely established that preservation of even a modest level of endogenous insulin production after clinical diagnosis is associated with reduced short- and long-term complications, providing a strong rationale for targeting immune pathways involved in the pathogenic process^3^.

The therapeutic landscape for T1D has recently changed with the regulatory approval of teplizumab (an Fc receptor-nonbinding, anti-CD3 monoclonal antibody) to prevent clinical T1D (stage 3) in individuals with preclinical T1D, who already show signs of dysglycemia (stage 2)^4,5^. Moreover, a growing number of immunotherapies are now being assessed at earlier stages of disease development, including in largely asymptomatic individuals identified on the basis of circulating autoantibodies (stage 1 T1D). Although immunotherapy in the preclinical phases of T1D can delay the need for insulin for a period of years with clear clinical benefit^6^, balancing risk and benefit is complex. To be appropriate for use at the early stages of disease, therapies should have minimal adverse effects, even with sustained administration, potentially over many years. Interventions that have shown efficacy in β-cell preservation to date include drugs that target large populations of T or B cells^7^. However, to reduce the long-term adverse effects of generalized immunosuppression, it would be preferrable to selectively target T cell subsets most closely responsible for β-cell destruction.

There has previously been conflicting evidence for the role of CD4 T helper cells producing IL-17 (T_H_17 cells) in T1D^8,9^. IL-23 is a key cytokine in the development of T_H_17 cells and *IL23A* has been identified as a candidate gene in recent T1D genetic association studies^10^. In murine models, IL-17 is upregulated in the pancreas and lymph nodes (LNs) early in disease^11,12^ and transfer of highly purified islet-specific T_H_17 cells can cause diabetes, although in some cases only after conversion to T helper cells that also secrete interferon-γ (T_H_17.1 cells) in vivo^13,14^. In mice, IL-17 could be a marker of pathogenicity rather than the mediator of islet damage because administration of anti-IL-17-blocking antibodies does not protect against disease^13,14^. In humans, T_H_17 and T_H_17.1 cells are upregulated in the blood, pancreas and LNs of individuals with T1D^15–18^. There is also evidence for a role for follicular T_H_ cells (T_FH_ cells, expressing CXCR5^+^ and PD-1^+^) under the control of IL-21 in T1D. This cell subset is upregulated in mouse pancreas and human blood in the context of T1D^16,19,20^ and, under the influence of IL-23 or IL-12, can secrete IL-17 along with IL-21 or interferon-γ (IFNγ). In addition, there is evidence that the combination of IFNγ and IL-17 exhibits direct cytokine-mediated killing of β-cells^18^.

Ustekinumab is a monoclonal antibody that binds the shared p40 subunit of IL-12 and IL-23. These two cytokines play a key role in the development of T_H_1 (IFNγ-secreting) and T_H_17 (IL-17 secreting) cells, respectively^21^. Ustekinumab has been licensed since 2009 for the treatment of psoriasis, psoriatic arthritis and inflammatory bowel disease, including for use in children as young as 12 years for some indications. More than 100,000 patients have been treated with ustekinumab and aggregated safety data from more than 20,000 patients have demonstrated an impressive safety profile, with sepsis rates consistently lower than anti-tumor necrosis factor (TNF) inhibitors, better preservation of vaccine responses^22–26^ and no increased cancer risk compared with anti-TNF inhibitors^27^.

A pilot study of ustekinumab in adult patients with newly diagnosed T1D (UST1D1) demonstrated a reduction in T_H_17.1 cells along with preliminary evidence of probable efficacy at doses used in inflammatory bowel disease^28^, but it did not have a placebo arm. In the present study, we present the results of a phase 2, multicenter, double-blind, randomized placebo-controlled trial of ustekinumab in children and adolescents within 100 days of diagnosis of T1D (the USTEKID study). We provide evidence for a key role for a small proinflammatory subset of T_H_17.1 cells in driving β-cell loss and demonstrate that targeting this subset by IL-12/IL-23 inhibition preserves C-peptide levels.

## Results

### Patient disposition

**Fig. 1:**
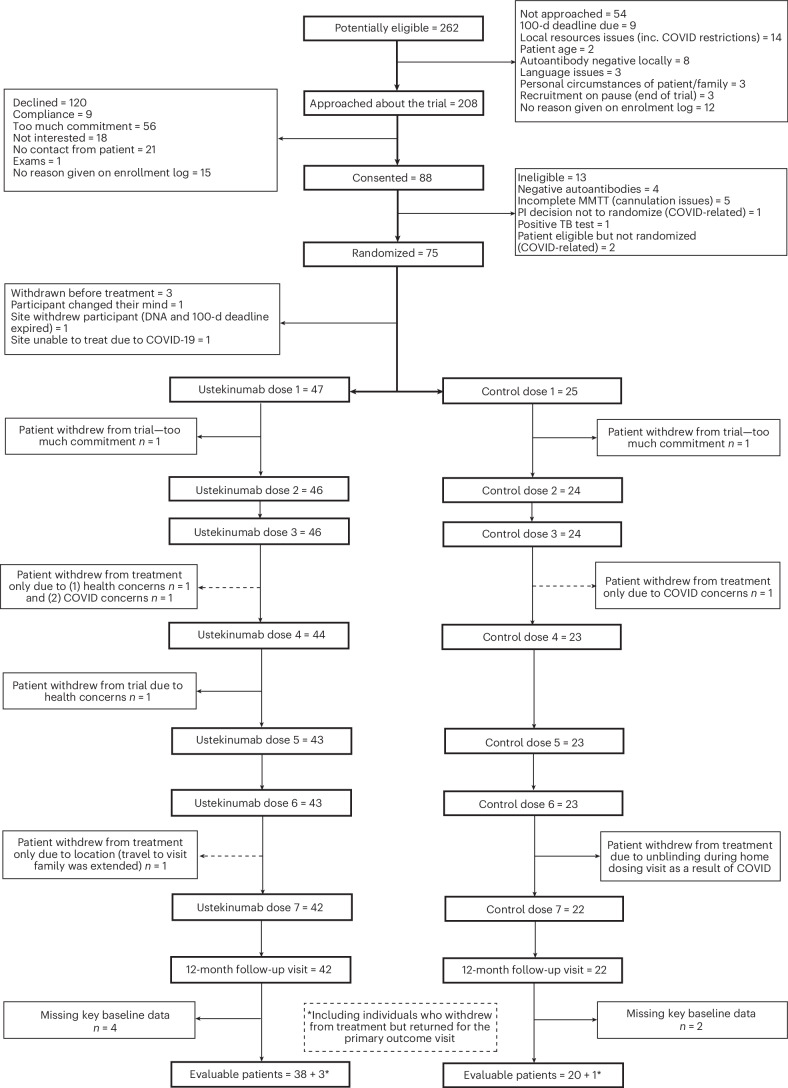
Consolidated-standards-of-reporting trials diagram showing screening and treatment allocation. Dashed lines indicate participants who withdrew from dosing but stayed in the trial and were included in the final analysis.

Of the 262 people who were identified as eligible for the study, we approached 208 and consented 88 participants (Fig. 1). Of these, 13 participants were not eligible for randomization due to negative β-cell autoantibody status (*n* = 4), incomplete mixed-meal tolerance test (MMTT) resulting from cannulation issues (*n* = 5), positive tuberculosis (TB) test (*n* = 1) or COVID-related issues (*n* = 3). A total of 72 participants were randomized in a 2:1 ratio (in favor of treatment) and allocated to two study arms. Three eligible participants withdrew before the first treatment and were replaced. Four participants withdrew from the trial after randomization (6%). A further four participants withdrew from treatment during the study but attended the primary endpoint assessment (week 52). In total, 68 participants attended the primary endpoint assessment (94%), of whom 64 were on treatment (89%). Six individuals were missing key baseline data required for the primary endpoint. Hence, 62 participants (86%) were included in the primary outcome measure analysis (41 in the ustekinumab group and 21 in the placebo group) (Fig. 1). The study was conducted from December 2018 to September 2022 (recruitment ended in October 2021 and the follow-up period in September 2022), part of which was during the COVID pandemic. Baseline characteristics are shown in Table 1. Study design allowed recruitment of both males and females. Results apply to both sexes. The ustekinumab and placebo groups were comparable in terms of sex, age, body mass index (BMI), ethnicity, baseline C-peptide area under the curve (AUC) and glycated hemoglobin (HbA1c). Participants attended centers with pediatric and adult diabetes teams with expertise in the management of T1D.

**Table 1:** Baseline characteristics of study participants

	Placebo (*n* = 25)	Ustekinumab (*n* = 47)
Sex, *n* (%)		
Male	16 (64)	27 (57)
Female	9 (36)	20 (43)
Age of diagnosis in years, mean (s.d.) (min., max.)	14.28 (1.65) (12, 18)	13.83 (1.74) (11, 18)
Age (categorical) (years), *n* (%)		
12–15	20 (80)	39 (83)
16–18	5 (20)	8 (17)
Age at screening in years, mean (s.d.) (min., max.)	15.0 (1.63) (12.46, 18.77)	14.49 (1.78) (12.18, 18.53)
Peak C-peptide level at screening (nmol l^−1^), *n* (%)		
0.2–0.7	5 (20)	8 (17)
>0.7	20 (80)	39 (83)
Ethnicity, *n* (%)		
White	20 (80)	39 (83)
Mixed race	1 (4)	4 (9)
Black or Black British	1 (4)	2 (4)
Asian or Asian British	1 (4)	1 (2)
Other ethnicity	2 (8)	1 (2)
Height (cm), mean (s.d.) (min., max.)	167.7 (10.57) (147.8, 189.8)	165.2 (10.21) (144.2, 184.0)
Weight (kg), mean (s.d.) (min., max.)	60.6 (13.70) (37.6, 96.6)	57.7 (13.85) (31.0, 97.8)
BMI (kg m^−2^), mean (s.d.) (min., max.)	21.3 (3.39) (15.5, 28.4)	21.0 (4.09) (14.9, 32.7)
Number of positive β-cell autoantibodies		
1	4	4
2	4	17
3	17	26
zBMI, mean (s.d.) (min., max.)	0.51 (0.97) (−1.58, 2.47)	0.40 (1.19) (−1.84, 3.02)
HbA1c, mean (s.d.) (min., max.)	48.6 (13.25) (8^a^, 74)	49.9 (10.15) (33, 80)
Daily insulin dose (units kg^−1^), mean (s.d.) (min., max.)	0.42 (0.19) (0.07, 0.80)	0.49 (0.32) (0.04, 1.39)
Duration of follow-up (months), mean (s.d.) (min., max.)	12.78 (0.32) (11.86, 13.31)	12.78 (0.98) (12.06, 18.16)
C-peptide AUC at screening (nmol l^−1^ min^−1^), mean (s.d.) (min., max.)	0.92 (0.48) (0.22, 1.87)	0.89 (0.50) (0.17, 2.75)

^a^One patient had a hereditary blood disorder; the impact of this data point was checked in a sensitivity analysis. Continuous data are displayed as arithmetic mean and s.d. Categorical data are displayed as number and percentage. *P* > 0.5 for all treatment comparisons. min., minimum; max., maximum.

### Primary outcome

**Fig. 2:**
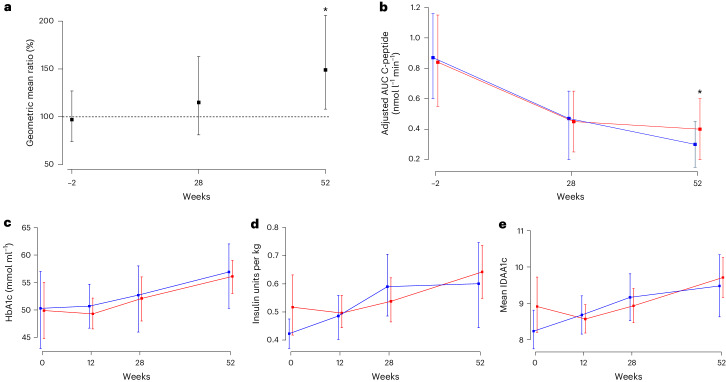
Primary and secondary metabolic outcome measures. **a**, Geometric ratio (with 95% CI) of intervention (ustekinumab) over control (placebo) over 52 weeks (ustekinumab group, *n* = 41; placebo group, *n* = 21). **b**, Adjusted AUC C-peptide (nmol l^−1^ min^−1^) over 52 weeks by treatment group (ustekinumab group, *n* = 41; placebo group, *n* = 21). **c**, HbA1c (mmol mol^−1^) over 52 weeks by treatment group (ustekinumab group, *n* = 44; placebo group, *n* = 20). **d**, Mean daily exogenous insulin use adjusted by body weight over 52 weeks by treatment group (ustekinumab group, *n* = 43; placebo group, *n* = 18). **e**, IDAA1c over 52 weeks by treatment group (ustekinumab group, *n* = 43; placebo group, *n* = 18). Measurements were performed at baseline, week 12 (HbA1c, insulin dose and IDAA1c only), week 28 and week 52. Data for primary outcome are presented as geometric mean ratios with 95% CIs and data for secondary outcomes are presented as arithmetic mean ratios with 95% CIs. One sample per subject was obtained at each study point. Subjects in the ustekinumab group are shown in red and those in the placebo group in blue.

As per the predetermined statistical analysis plan, the C-peptide AUC in a 2-h MMTT was compared between the ustekinumab group and the placebo group over each time point (weeks 28 and 52) and adjusted for sex, baseline values of age, C-peptide AUC, HbA1c and exogenous insulin dose with the use of analysis of covariance (ANCOVA) models, the primary outcome being assessed at 52 weeks. Ustekinumab was associated with a difference of 49% higher C-peptide AUC in the treatment group at week 52 (ustekinumab 0.45 nmol l^−1^ min^−1^ versus placebo 0.30 nmol l^−1^ min^−1^, geometric ratio of ustekinumab:placebo 1.49 (95% confidence interval (CI) 1.08, 2.06); *P* = 0.02) (Fig. 2a,b and Supplementary Table 1). It is interesting that the effect of ustekinumab was delayed, despite sustained drug levels, with an initial and equivalent decline of C-peptide being observed in both groups until week 28 (ustekinumab 0.49 nmol l^−1^ min^−1^ versus placebo 0.42 nmol l^−1^ min^−1^, geometric mean ratio of ustekinumab:placebo was not significantly different at 1.15 (95% CI 0.81, 1.63); *P* = 0.45) after which the groups separated.

### Secondary outcomes

HbA1c levels rose across both groups from 50 mmol mol^−1^ at baseline to 56 mmol mol^−1^ at week 52 (Fig. 2c). No difference was seen in HbA1c between the groups (mean difference between ustekinumab and placebo at week 52 = −0.83, 95% CI of the difference = −7.2, 5.55, *P* = 0.15). Exogenous insulin use increased from baseline to week 52 in both groups (0.42 units kg^−1^ to 0.63 units kg^−1^ in the control group; 0.51 units kg^−1^ to 0.63 units kg^−1^ in the ustekinumab group) with no difference between the groups after adjustment for baseline factors (mean difference between groups at week 52 = 0.04, 95% CI of the difference = −0.13, 0.21, *P* = 0.38) (Fig. 2d). Insulin dose-adjusted HbA1c (IDAA1c) also increased in both groups (8.23% to 9.46% in the control group and 8.90% to 9.69% in the ustekinumab group) with no difference between the groups (mean difference between groups at week 52 = 0.23, 95% CI of the difference = −0.79, 1.24, *P* = 0.65) (Fig. 2e).

No significant difference was seen in between the groups with regard to other secondary outcomes: glycemic variability parameters downloaded from the blood glucose monitoring system (CGM), for example, percentage of time >10 mmol l^−1^ and >13.9 mmol l^−1^ (Supplementary Table 2) and percentage time hypoglycemic (<3.0 mmol and <4.0 mmol; Supplementary Table 3), number of clinical hypoglycemic events (Supplementary Table 4a–c) and the Hypoglycaemia Fear Survey (HYPOFEAR), Diabetes Treatment Satisfaction Questionnaire (DTSQ) and Pediatric Quality of LIfe Inventory (PedsQL) completed by participants (Supplementary Table 5a) and their parent/carer (Supplementary Table 5b).

### Safety and drug levels

**Extended Data Fig. 1:**
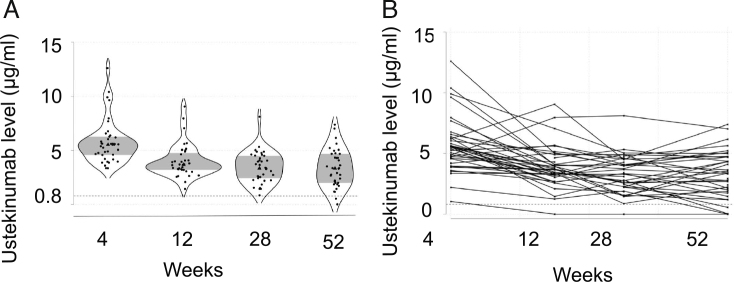
Ustekinumab levels over the study period. (**a**) Violin plot of Ustekinumab levels; (**b**) Connected line plot of Ustekinumab levels. Individual data points shown, shaded area represented interquartile range. Reference line at 0.8 μg/ml.

Ustekinumab was very well tolerated with no serious adverse events considered to be treatment related. Frequency and type of side effects were comparable between the ustekinumab and the placebo groups (Supplementary Table 6a–c). With the exception of one participant at week 28, all participants who still received the active drug had ustekinumab levels above a reported therapeutic level of 0.8 μg ml^−1^ (ref. ^29^) for the duration of the study, only for those on treatment (Extended Data Fig. 1).

### Exploratory outcomes

**Fig. 3:**
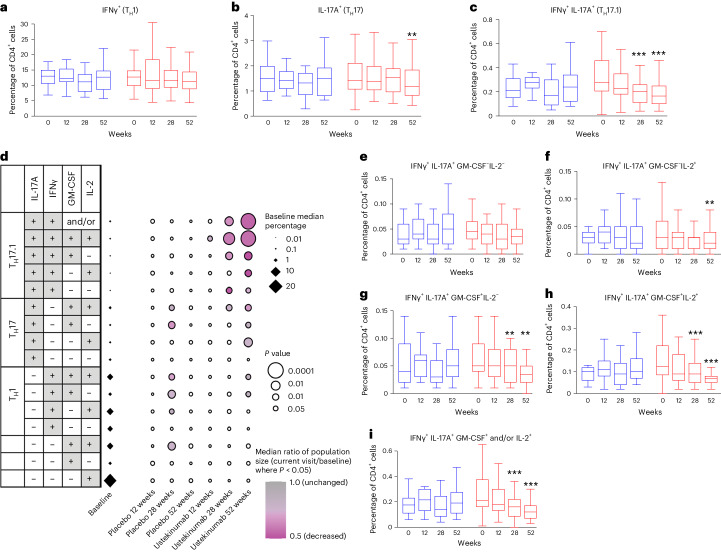
Analysis of the frequency of cytokine-producing CD4 T cell subsets in individuals treated with ustekinumab and placebo. **a**–**c**, Box plots of frequencies of CD4^+^ T cells producing IFNγ (**a**; T_H_1 cells), IL-17A (**b**; T_H_17 cells) and IFNγ and IL-17A (**c**; T_H_17.1 cells) (week 28, *P* = 0.001; week 52, *P* < 0.0001). **d**, Dot plot of changes in cytokine-producing CD4 T cell subsets during treatment. The ratio of each population was calculated as current visit/baseline for each participant for every time point for which they had data. Statistical significance was determined using the Kruskal–Wallis rank test and circle size was scaled by the *P* value, with more significant *P* values represented by larger circles. Data points with *P* < 0.05 are colored by the median ratio of population size (gray = 1 (unchanged) to purple = 0.5 (halved)). Data points with *P* > 0.05 are colored white. The baseline median percentage of each population is represented by scaled black diamonds. **e**–**i**, Box plots of frequencies of CD4^+^ T cells in various combinations of cytokines as indicated: IL-17A^+^IFNγ^+^GM-CSF^−^IL-2^−^ (**e**), IL-17^+^IFNγ^+^GM-CSF^−^IL-2^+^ (**f**) (week 52, *P* = 0.005), IL-17A^+^IFNγ^+^GM-CSF^+^IL-2^−^ (**g**) (week 28, *P* = 0.002; week 52, *P* = 0.001), IL-17A^+^IFNγ^+^GM-CSF^+^IL-2^+^ (**h**) (week 28, *P* = 0.001; week 52, *P* < 0.0001) and IL-17A^+^IFNγ^+^GM-CSF^+^ and/or IL-2^+^ (**i**) (week 28, *P* = 0.001; week 52, *P* < 0.0001). For **a**–**c** and **e**–**i** ^**^P < 0.01, ^***^P < 0.001. The line represents the median, the box the interquartile range (IQR) and the whiskers all data points within 1.5× the IQR of the nearer quartile; outliers are excluded. Forty-four participants in the ustekinumab group and twenty-one in the placebo group are included in the analysis presented in **a**–**c**. Forty-four participants in the ustekinumab group and nineteen in the placebo group were included in the analysis presented in **d**–**i**. One sample per subject was obtained at each study point. Statistical significance was determined using two-sided Wilcoxon’s matched-pairs, sign-rank test (**a**–**c** and **e**–**i**). Ustekinumab was labeled as red and placebo as blue.

We collected whole blood and assessed cytokine production in real time, focusing on cytokines produced by T cell populations targeted by ustekinumab and/or associated with T1D pathogenesis. Whereas we did not observe a reduction in the frequency of CD4 T cells producing IFNγ, in the ustekinumab group, there was a significant reduction in cells producing IL-17A (T_H_17 cells) and those producing both IL-17A and IFNγ (T_H_17.1 cells) (Fig. 3a–c). Of note, this decrease was observed at 28 weeks (T_H_17.1 cells) and 52 weeks (T_H_17 and T_H_17.1 cells) but not at 12 weeks. To further dissect how ustekinumab affected circulating CD4 T cells, we analyzed the change in frequency of CD4 T cells producing combinations of IL-17A, IFNγ, granulocyte–macrophage colony-stimulating factor (GM-CSF) and/or IL-2. This analysis revealed that the most pronounced effect of ustekinumab was seen in T_H_17.1 cells that also produced GM-CSF and/or IL-2 (Fig. 3d–i). Although it represented only <0.1% of the total proportion of CD4^+^ T cells, this rare circulating subset producing all four cytokines (IFNγ^+^, IL-17A^+^, GM-CSF^+^, IL-2^+^) showed a modest reduction as early as 12 weeks and a highly significant (*P* < 0.001) reduction at weeks 28 and 52 after the start of therapy (Fig. 3d,h). In contrast, T_H_17.1 cells that did not produce either GM-CSF or IL-2 showed little effect of treatment (Fig. 3d,e).

**Extended Data Fig. 2:**
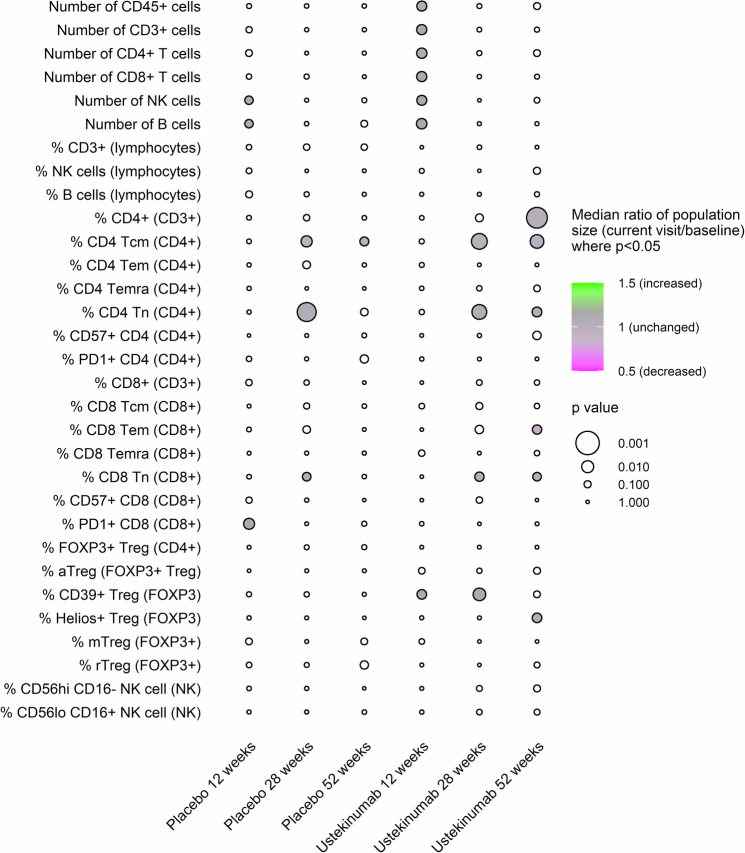
Dotplot of immune cell populations during treatment, determined by flow cytometry. Populations were defined as shown in Supplementary Material Fig. 1. Parent population are indicated in parentheses. The ratio of each population was calculated as current visit/baseline for each participant for every timepoint for which they had data. Statistical significance was determined using a two-sided Wilcoxon test and circle size is scaled by p value, with more significant p values represented by larger circles. Data points with p < 0.05 are coloured by the median ratio of population size (grey=1 (unchanged) to purple 0.5 (halved) and green 1.5 (increased by 50%)). Data points with p > 0.05 are coloured white.

We also examined the number and frequency of the main circulating leukocyte populations using multidimensional flow cytometry. We did not observe any consistent, treatment-related changes in the absolute number or frequency of total T cells, CD4 T cells, CD8 T cells or natural killer (NK) cells or a change in the frequency of B cells, CD4 and CD8 T cell naive/memory subsets, NK cell subsets or FOXP3^+^ regulatory T cells (T_reg_ cells) (Extended Data Fig. 2)

**Fig. 4:**
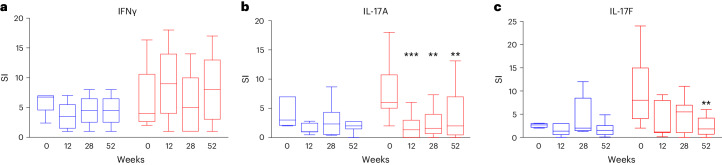
Box plot of cell producing IL-17A in response to stimulation with proinsulin in individuals treated with ustekinumab and placebo. **a**, IFNγ response. **b**, IL-17A response (week 12, *P* = 0.0003; week 28, *P* = 0.006; week 52, *P* = 0.002). **c**, IL-17F response (week 52, *P* = 0.002). SI, mean no. of spots in proinsulin-stimulated well individuals/mean number of spots in unstimulated well individuals. Individuals with a baseline SI < 2 were removed. ^**^P < 0.01, ^***^P < 0.001. The line represents the median, the box the IQR and the whiskers the 95% range. Nineteen participants in the ustekinumab group and eight in the placebo group were included in the analysis presented in **a.** Twenty-one participants in the ustekinumab group and eleven in the placebo group were included in the analysis presented in **b**. FIfteen participants in the ustekinumab group and eight in the placebo group were included in the analysis presented in **c**. One sample per subject was obtained at each study point. Statistical significance was determined using two-sided Wilcoxon’s matched-pairs, sign-rank test. Ustekinumab was labeled as red and placebo as blue.

To assess the effect of treatment on islet-specific immune responses, we measured secretion of IFNγ, IL-17A and IL-17F in cryopreserved peripheral blood mononuclear cells (PBMCs) stimulated with proinsulin using a three-color cytokine FluoroSpot assay. At baseline, 30 of 67, 33 of 67 and 25 of 67 subjects had a substantial response to proinsulin (defined as a stimulation index (SI) ≥ 2) for secretion of IFNγ, IL-17A and IL-17F, respectively. In these individuals, the frequency of IL-17A and IL-17F cytokine-producing cells was significantly reduced in comparison to baseline in the ustekinumab group from 12 weeks (IL-17A) and at 52 weeks (IL-17F). In contrast, we observed no significant change in the proinsulin-stimulated IFNγ response in either group at any time point (Fig. 4).

### Post hoc analysis

#### Relationship between C-peptide levels and immune response to treatment

**Fig. 5:**
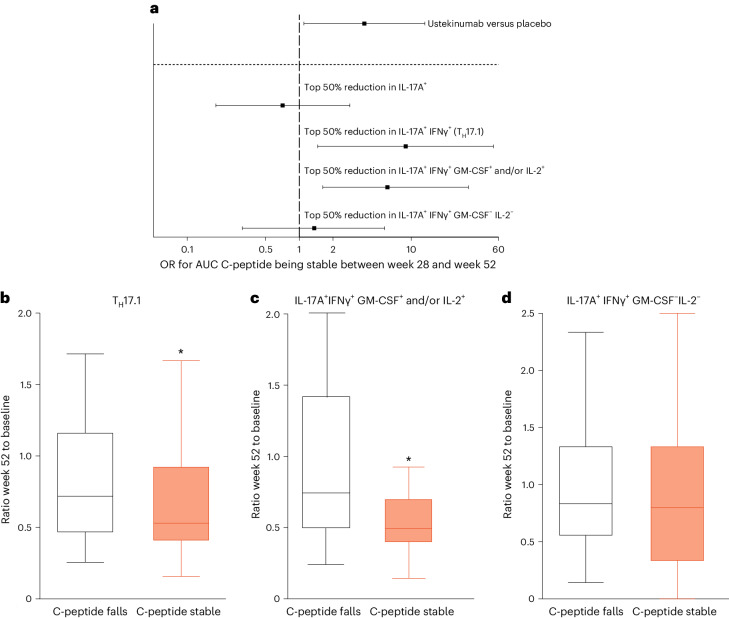
Relationship between change in immune parameters and primary metabolic outcome. **a**, OR (with 95% CI) of having a stable or increasing C-peptide level between weeks 28 and 52. Above the horizontal dashed line is the whole-study group comparing placebo and ustekinumab treated. Below the dashed line it compares those on ustekinumab stratified based on the change in immune subsets (from baseline to week 52) as indicated. The vertical dotted line denotes an OR of 1 (no effect). The square represents the OR points estimate and the lines represent the 95% CI. **b**–**d**, Box plots of the change in immune population (expressed as the ratio of the frequency at week 52 relative to baseline) stratified by the stability of C-peptide between weeks 28 and 52. The line represents the median, the box the IQR and the whiskers all data points within 1.5× the IQR of the nearer quartile; outliers are excluded. A value of <1 indicates a reduction in the immune population in response to treatment (**b**) change in T_H_17.1 cells at week 52 versus baseline (*P* = 0.03), change in IL-17A^+^IFNγ^+^GM-CSF^+^ and/or IL-2^+^ at week 52 versus baseline (*P* = 0.02) (**c**) and change in IL-17A^+^IFNγ^+^GM-CSF^−^IL-2^−^ at week 52 versus baseline (**d**). ^*^P < 0.05 for odds of having a lower ratio. From the ustekinumab group, 41 participants and, from the placebo group, 21 participants were included in the analysis presented in **a**. Analysis presented in **b**–**d** included 34 ustekinumab-treated partcipants. Statistical significance was determined by using logistic regression for the odds of having a stable C-peptide at week 52 adjusted for age. gender, baseline C-peptide and week 28 C-peptide in the analysis presented in **b**–**d**.

To determine the relationship between immune response to treatment and clinical outcome, we investigated a post hoc analysis of whether participants in the ustekinumab group who showed a larger reduction in cytokine-secreting cells (that is, ‘high immune responders’) also had better C-peptide preservation. To control for the delayed response in the effect of ustekinumab, we assessed the odds of the level of C-peptide being stable between weeks 28 and 52 (that is, unchanged or increasing during this period) in groups stratified by treatment or top 50% immune response (defined as top 50% reduction in immune cells of interest) between baseline and week 52. As shown in Fig. 5a (above dashed line), being randomized to ustekinumab is significant for maintaining C-peptide stability with an odds ratio (OR) of 3.81 (95% CI = 1.10, 13.2, *P* = 0.03) in agreement with the primary outcome measure as described above. However, this OR is increased to 8.80 (95% CI = 1.46, 52.8, *P* = 0.01) in individuals with the highest reduction in T_H_17.1 cells, but this was not observed in individuals on placebo with OR of 1.67 (95% CI = 0.13, 20.6, *P* = 0.69). Furthermore, dissection of the T_H_17.1 cell population, based on co-secretion of other cytokines, revealed that maintaining stabile C-peptide was associated with a reduction in cells secreting a combination of IL-17A and IFNγ and either GM-CSF and/or IL-2 (OR = 6.12 (95% CI = 1.16, 32.3, *P* = 0.03)) but not associated with the reduction in cells secreting IL-17A and IFNγ in the absence of either GM-CSF and/or IL-2 (Fig. 5, below dashed line). To confirm this association, we also stratified ustekinumab-treated individuals based on their clinical outcome (that is, C-peptide responders, defined as having C-peptide that is stable between weeks 28 and 52) and examined the relative reduction in immune populations in these groups (Fig. 5b–d). This analysis confirmed the association between C-peptide retention and treatment-induced change in immune response with a significant reduction in T_H_17.1 cells in those with stable C-peptide (Fig. 5b) and that this was specific to T_H_17.1 cells that co-secrete GM-CSF and/or IL-2 (Fig. 5c,d).

### Sensitivity analysis

Sensitivity analyses were performed to confirm robustness of the conclusions about the analysis of the primary outcome to protocol deviations. The exclusion of one participant who accidentally became unblinded, one participant whose primary outcome visit was delayed by 6 months and one with a hereditary red cell disorder affecting HbA1c had no effect on the primary outcome. Hence, the model for analyzing the primary outcome was robust to small numbers of people with some protocol deviations and extreme values in key covariates.

Multiple imputation was performed as a sensitivity check for the impact of missing data on the primary analysis. After imputation 10×, the geometric ratio of ustekinumab to control changed to 1.36 (95% CI = 0.81, 1.63; *P* = 0.27) and did not reach statistical significance. The conclusion about the treatment group differences might therefore be affected by missing data.

Sensitivity checks were also performed to check sensitivity of the conclusions to non-normality of the data distribution for key secondary outcomes. Both linear and logarithmic data were built for HbA1c, exogenous insulin use and IDAA1c. The results were similar and, therefore, the simpler linear models were reported.

## Discussion

The main conclusion from the present study is that ustekinumab demonstrated a high safety profile and positive effect on β-cell preservation in children and adolescents with recently diagnosed T1D by targeting the IL-12/IL-23 pathway. This provides the first prospective randomized controlled trial evidence for a pathogenic role of T_H_17 cells in T1D, confirming preliminary data from the preceding pilot study^28^.

Our exploratory mechanistic data suggest that a pathogenic subset of T_H_17 cells representing around 0.1% of the circulating CD4 T cell population, characterized by co-expression of IL-17A, IFNγ, GM-CSF and IL-2, plays a key role in the loss of β-cell function. Ustekinumab reduced this cell population and preserved C-peptide levels. The highly selective nature of the T cell modulation produced by ustekinumab reduces its impact on other parts of the immune system and underlies its favorable adverse event safety profile seen in the present study and over its extensive 14-year clinical use in other conditions^22–26^. There are several findings in our exploratory mechanistic analysis that are of particular interest.

First, the phenotype of the cells that associated with a favorable response to treatment is notable. T_H_17 cells have been implicated in the pathogenesis of multiple inflammatory and autoimmune diseases; however, they can also play an important role in tissue protection and homeostasis^30^. Evidence from in vitro differentiation and mouse lineage-tracing experiments suggests that IL-17-secreting cells represent a heterogeneous and plastic population of cells that have a functional phenotype influenced by their cytokine and metabolic environment^31–34^. In these studies, nonpathogenic (or homeostatic) subtypes of T_H_17 cells are characterized by secretion of immunoregulatory cytokines such as IL-10, whereas pathogenic T_H_17 cells are characterized by co-secretion of proinflammatory cytokines, IFNγ (T_H_17.1 cells) and GM-CSF. Ustekinumab targets IL-12 and IL-23. Multiple lines of evidence from mouse models demonstrate that IL-23 is a crucial factor in the polarization of T_H_17 cells toward a pathogenic profile^30,34–37^. In human autoimmune diseases, including psoriasis, multiple sclerosis and rheumatoid arthritis, pathogenic T_H_17 cells appear to play a key role, being present at sites of pathology and correlating with disease activity^38–40^.

Previous studies have identified islet-specific T_H_17.1 cells that produce GM-CSF in patients with T1D but a direct link with β-cell destruction has been lacking^41^. Our observation that the efficacy of treatment with ustekinumab is specifically associated with reduction of T_H_17.1 cells secreting GM-CSF provides strong evidence for a role of these cells in driving β-cell destruction. It is interesting that, in multiple sclerosis, GM-CSF seems to play an active role in initiation of central nervous system inflammation^42^ because autoreactive T cells that lack GM-CSF fail to initiate neuroinflammation despite IL-17 and IFNγ production^43^.

Our results are also consistent with a recently published biomarker analyses of a clinical trial of alefacept (LFA3-IgG) in patients with new-onset T1D, which demonstrated that islet antigen-reactive CD4^+^ T cells were enriched in T_H_17.1 cell phenotypes in people with T1D, including cells co-expressing GM-CSF, IL-2, IFNγ and IL-17. These cells were inversely correlated with C-peptide preservation in treated individuals^44^, supporting the hypothesis that targeting this population reduces β-cell loss. The surface phenotype of the T_H_17.1/GM-CSF^+^/IL-2^+^ CD4 cells modulated in our study remains uncertain. However, there may be overlap with a subgroup of T_FH_ cells (typically CXCR5^+^, PD-1^+^ICOS^+^) that also seem to be relevant to T1D^45,46^ and are impacted by ustekinumab^47^.

Second, in contrast to other less targeted immunotherapies for T1D, the effect of treatment on both the immune system and β-cell function were substantially delayed. Slowing of β-cell loss did not become apparent in the treated group for 6–12 months and, in keeping with this, although the effect on T cell subsets was apparent at 3 months, it did not become maximal until 6–12 months. Most other immunotherapy studies in T1D have shown benefit early with a lesser effect beyond 6 months, which means that the effect that we observed may have been missed if the primary endpoint had been at 6 months rather than 12 months^47^. However, this time course in metabolic and exploratory mechanistic findings in the present study was consistent with the adult pilot study in which slowing of the loss of β-cell function and the maximal effect on T_H_17.1 cells was also not apparent until 6–12 months^28^. In addition, although there was no control group in this pilot, subjects losing C-peptide more slowly were found to have a greater reduction in T_H_17.1 cells^28^. The delayed therapeutic effect may result partially from the fact that ustekinumab impairs the polarization of T_H_17 cells toward a pathogenic phenotype (via IL-23 inhibition), but may have less effect on already polarized cells. This possibility is consistent with maximal T cell changes not being achieved for 12 months. However, this differs from the timing of the clinical impact seen in psoriasis, psoriatic arthritis or inflammatory bowel disease, where almost maximal clinical improvements are apparent by 16 weeks^48–50^. It is therefore possible that the delayed effect is also the result of a requirement for other changes occurring downstream of the impact on T_H_17.1 cells before β-cell loss in T1D. Notably, even after 52 weeks of sustained therapeutic levels of ustekinumab, the reduction of T_H_17.1 cells and T_H_17.1/GM-CSF^+^/IL-2^+^ cells was only partial, representing approximately a 50% reduction from baseline (Fig. 3). Despite IL-12 inhibition, no significant effect of ustekinumab was seen on the T_H_1 cells (IFNγ-secreting cells), consistent with the findings in patients with inflammatory bowel disease treated with ustekinumab^51,52^.

Third, consistent with the adult pilot study, we show that ustekinumab was able to reduce the frequency of islet antigen-specific T cells, as seen by proinsulin-stimulated FluoroSpot. This effect was seen at an earlier time point than the reduction in cytokine secretion after polyclonal stimulation, suggesting that the generation of islet-specific T_H_17 cells is still an active process even after diagnosis. Consistent with the results of the polyclonal stimulation, ustekinumab targeted IL-17-secreting cells, including both IL-17A and IL-17F, but islet-specific T cell-secreting IFNγ did not seem to be reduced (Fig. 4a).

From a clinical perspective, the reduction in β-cell destruction did not translate into a significant effect on other metabolic parameters (HbA1c, time in range on CGM and IDAA1c) during the timeframe of the study. However, the study was underpowered to detect such changes, which typically require 2–3× greater sample sizes^53^. Furthermore, the delayed onset of action meant that >40% of C-peptide production was lost before the preservation effect of the intervention became apparent, which is likely to impact any metabolic benefits and may also indicate that longer-term treatment may be needed to see any improvement in other metabolic measures.

The present study does provide a rationale to attempt the use of ustekinumab in a prevention study as a next step. The delayed action of the drug in reducing the target immune population in the new-onset population in whom β-cell destruction is occurring at such a rapid pace suggests that this therapeutic may be better suited to use at an earlier stage of disease such as stage 1 or stage 2 T1D, where a delay in clinical efficacy may be more acceptable. Indeed, the well-established safety profile of ustekinumab in chronic use, consistent with its highly targeted effects on pathogenic T_H_17 cell subsets, as well as the low burden of subcutaneous dosing every 2 months, make it particularly attractive for use in preclinical disease. Alternatively, ustekinumab could be used to prolong the effect of drugs that have a major early effect such as teplizumab or ATG^54^.

Another approach in a future efficacy study would be the use of alternative biologics in clinical use that target T_H_17 cells, including drugs targeting the IL-23 receptor specifically via the p19 subunit (guselkumab, risankizumab and tildrakizumab) and others directly targeting IL-17 (ixekizumab and secukinumab) or the IL-17 receptor (brodalumab). In inflammatory bowel disease and psoriasis, these agents appear to be more rapid acting and more effective, suggesting that they might be considered in T1D^55,56^. However, anti-IL-17 was ineffective in preventing T1D in NOD–SCID (nonobese diabetic/severe combined immunodeficiency) mice^13,14^ and we cannot rule out a relevant effect of the IL-12-blocking component of ustekinumab to stop a diversion to the T_H_1 cell pathway after T_H_17 cell inhibition or effects on other T cell subsets such as mucosa-associated invariant T cells^57^, which may play a role in T1D^58^. A final approach that could be considered is the combination of ustekinumab with a synergistic agent that has already proved effective in T1D efficacy studies (for example, baricitinib or T_reg_ cell enhancement).

The strengths of our data include the randomized double-blind nature of our study, including blinding of laboratory staff and the use of fresh blood flow cytometry, along with strict quality assurance procedures in the assays^59^.

Study limitations include that the T cell assays were exploratory rather than primary endpoints of the study. Importantly, no adjustment was made for multiple testing, although the level of significance (*P* < 0.001) and the clustering of significant outcomes around small but overlapping populations provides strong support for the conclusion (Fig. 3d). Replication is nevertheless required and the UST1D2 study in adults using a similar protocol and harmonized T cell analyses is currently ongoing (NCT03941132). In addition, the study was underpowered to detect changes in metabolic parameters, especially as the number of complete datasets at week 52 for the primary endpoint was less than anticipated in our power calculation (62 versus 66 patients). This was the result partly of a drop-out of participants in the trial (*n* = 4), but also of missing baseline data (*n* = 6) required for the baseline adjustments prespecified in the primary outcome analysis.

Our exploratory data suggest a role for a subset of T_H_17 cells in T1D that can be modulated at low risk by IL-12/IL-23 inhibition, with benefits on β-cell preservation. This represents a significant advance in treatment precision^60^. Further clinical trials are required to define whether IL-23 or IL-17 inhibition alone can replicate or enhance this effect and to define the role of T_H_17 cell modulation in the expanding list of options for reducing or delaying the need for insulin in T1D^1^.

## Methods

### Ethics statement

The present study was carried out with the approval of the UK Research Ethics Service (approval received on 18 September 2018 from Wales Research Ethics Committee (REC 3) reference 18/WA/0092) and UK Medicines and Healthcare products Regulatory Agency (MHRA) for Clinical Trial Authorisation (approval received on 26 June 2018). Written informed consent or assent was obtained from all participants. The trial was conducted in compliance with the principles of the Declaration of Helsinki (2013) and the principles of good clinical practice and in accordance with all applicable regulatory requirements including, but not limited to, the UK Policy Framework for Health and Social Care Research 2017 and the Medicines for Human Use (Clinical Trial) Regulations 2004, and subsequent amendments.

Participants were given up to £100 as an expression of gratitude for their commitment to the study.

### Study design

The study was a phase 2, multicenter, double-blind, randomized, placebo-controlled trial of safety and efficacy of ustekinumab in preserving endogenous insulin production measured by mixed-meal-stimulated, 2-h plasma C-peptide AUC at week 52 in children and adolescents aged 12–18 years within 100 d of diagnosis of T1D^61^.

The trial was conducted in 16 pediatric and adult diabetes research centers in the United Kingdom: Royal London Hospital, London; Royal Alexandra Children’s Hospital, Brighton; Countess of Chester Hospital, Chester; East Lancashire Hospitals NHS Trust, Burnley; Evelina London Children’s Hospital, London; Royal Devon and Exeter Hospital, Exeter; St James’ Hospital, Leeds; Leicester Royal Infirmary, Leicester; Norfolk and Norwich University Hospitals, Norwich; St George’s University NHS Trust, London; University College London, London; University Hospital of Wales, Cardiff; Noah’s Ark Children’s Hospital, Cardiff; Swansea Bay University Health Board, Swansea; Ninewells Hospital, Dundee; and Royal Aberdeen Children’s Hospital, Aberdeen.

The investigational medicinal product (IMP) was ustekinumab, a fully human immunoglobulin (Ig)G1κ monoclonal antibody supplied by the marketing authorization holder Janssen-Cilag (EU/1/08/494/002). It was supplied as sterile, single-use, 2-ml glass vials containing 0.5 ml of solution with 45 mg of ustekinumab for injection. Saline in the form of sodium chloride 0.9% w:v solution for injection was used as placebo. Participants were given ustekinumab/placebo (2:1) subcutaneously at weeks 0, 4, 12, 20, 28, 36 and 44, with the dose depending on their body weight (2 mg per kg body weight if the participant was ≤40 kg and 90 mg if >40 kg), and were followed for 12 months after the first dose.

The main inclusion criteria were as follows: 12–18 years of age; clinical diagnosis of immune-mediated T1D as defined by the American Diabetes Association (ADA); started on insulin within 1 month of diagnosis; an interval of ≤100 days between the confirmed diagnosis (defined as date of first insulin dose) and the first planned dose of the IMP; written and witnessed informed consent/assent to participate; evidence of residual functioning β-cells (peak serum C-peptide level >0.2 nmol l^−1^ in MMTT); positive of at least one islet autoantibody (glutamic acid decarboxylase (GADA), insulinoma-associated antigen 2A (IA-2A) and zinc transporter protein 8 (ZnT8)); and body weight <100 kg.

The main exclusion criteria were: use of immunosuppressive or immunomodulatory therapies including systemic steroids; use of any hypoglycemic agents other than insulin for >6 weeks at any time before trial entry; prior exposure to ustekinumab within 3 months of the first dose of the IMP; prior allergic reaction, incuding anaphylaxis to any component of the IMP; notably abnormal laboratory results during the screening period other than those due to T1D; use of inhaled insulin; known alcohol or drug abuse; evidence of active hepatitis B, hepatitis C, human immunodeficiency virus (HIV) or considered by the investigator to be at high risk for HIV infection; immunization with live vaccines 1 month before trial entry; history of current or past active TB infection; latent TB; substantial systemic infection during the 6 weeks before the first dose of the IMP; and breastfeeding, pregnancy or unwillingness to comply with contraceptive advice and regular pregnancy testing throughout the trial.

Safety laboratory measures of hematological indices, liver function, thyroid-stimulating hormone, urea, creatinine, calcium, lipid levels and Ig levels and urine assessments (pH, blood, protein by dipstick analysis, laboratory analysis for albumin:creatinine ratio) were performed throughout the study. HIV and hepatitis B and C and TB testing were performed at screening. Adverse events were reported by participants and reviewed by the site principal investigator (PI) at all visits.

The trial oversight was performed by a trial steering committee and an independent data safety monitoring board.

International Standard Randomised Controlled Trial Number Registry: registration no. ISRCTN 14274380.

### Assays

#### β-Cell function

##### MMTT

Ensure Plus (Abbott Nutrition; 6 ml kg^−1^ (max. 360 ml)) was used as a mixed-meal stimulant of β-cell production, in the standard MMTT as previously described^62^. The MMTTs were carried out after an overnight fast at −2, 28 and 52 weeks. Plasma samples for C-peptide and glucose were collected in EDTA and fluoride oxalate bottles, respectively, at 0, 15, 30, 60, 90 and 120 min. Plasma samples were stored at −20 °C and transported on dry ice in batches. Serum C-peptide was measured using an immunochemiluminometric assay (Invitron, cat. no. IV2-004). The detection limit and intra- and interassay coefficients of variation were 0.005 nmol l^−1^, <5% and <8%, respectively.

#### Glycemic control

##### Blood glucose monitoring

All participants were provided with an Abbott FreeStyle Libre blood glucose monitoring system (CGM). Participants were expected to wear a sensor for at least 2 weeks before each study visit and were advised to read their measurements at least 4–7× a day. Anonymized data were sent electronically to the trial office.

##### HbA1c

HbA1c was tested in the local NHS laboratories of the study sites to guide clinical care. The HbA1c target value was set according to 2015 National Institute for Health and Care Excellence (NICE) guidelines (available at www.nice.org.uk/guidance/ng182015) in agreement with the participant and their clinical care team. An additional blood sample was taken at weeks 0, 12, 28 and 52 for measurement of HbA1c using a high-performance liquid chromatography method in a central laboratory.

##### Daily insulin dose

Mean daily insulin use was calculated over 7 consecutive days during the 2 weeks preceding all visits and participants were asked to record all insulin usage in their daily diary during those 2 weeks. This value was calculated in international units of IU kg^−1^ d^−1^. Where data from consecutive days were not available, the 3 d closest together were used.

##### Hypoglycemia

Participants were advised by the research staff to record in a trial diary any symptoms possibly related to hypoglycemia and their timing to allow later comparison with glucose monitoring data. A finger-prick blood glucose was recorded in the diary any time hypoglycemic symptoms occurred, even if the glucose monitor sensor was also being worn.

The PI or delegate categorized all hypoglycemic events recorded in the diary according to the ADA guidelines^63^.

The number of severe hypoglycemic events was recorded at weeks 78 and 104 to cover the period since the previous data collection time point. Severe was defined as:

1. Admission to hospital;
2. An ambulance being called but no transfer to hospital was needed;
3. Being given glucagon but no ambulance was called and no admission to hospital was needed;
4. Convulsions (fits) or loss of consciousness.

#### Body weight and BMI

Body weight and height were recorded at site visits, and the most recent weight recorded was used to calculate drug dosages for forthcoming treatment visits. The BMI was calculated as standard: weight (kg)/(height (m))^2^.

#### Patient and parent-reported outcome measures

Quality of life for participants and their parent/carer was assessed at screening and weeks 28 and 52 by validated questionnaires: HYPOFEAR^64,65^; DTSQ for inpatients^66^; and PedsQL (generic core scale^67,68^ and diabetes-specific^69,70^ modules).

The questionnaires were completed during the latter stages of the MMTT while the participant and parent were waiting for the end of the test. Participant and parent were encouraged not to discuss their responses with each other.

#### Immunological assays

##### β-Cell autoantibody measurements

Anti-GADA, anti-IA-2A and anti-ZnT8A were measured by ELISA (GDE/96, IAE/96/2, ZnT8/96; RSR Ltd) according to the manufacturer’s instructions. Positive cut-off values were ≥5, ≥7.5 and ≥15 U ml^−1^ for GADA, IA-2A and ZnT8A, respectively. Detection limit for GADA was 0.57 U ml^−1^, for IA-2A 0.95 U ml^−1^ and for ZnT8A 1.2 U ml^−1^.

##### Flow cytometry

Intracellular cytokine staining: 100 μl of fresh sodium heparin blood were stimulated with phorbol 12-myristate 13-acetate–ionomycin for 3 h using the DURActive1 DuraClone tubes (Beckman Coulter), according to the manufacturer’s instructions. After the end of incubation, the blood was stained with Live Dead Yellow dye (Invitrogen) at room temperature for 20 min. The blood was then lysed, fixed and permeabilized using the PerFix-nc kit (Beckman Coulter), according to the manufacturer’s instructions. The cells were then transferred and stained in the dark at room temperature for 45 min using the DuraClone IF T_H_ cell tube (Beckman Coulter), with the addition of drop-in antibodies targeting GM-CSF, IL-2 and CD8-PC5 (all diluted 1:50) as shown in Supplementary Table 7. The cells were then washed and acquired in the Beckman Coulter Navios flow cytometer. Flow data were analyzed using Kaluza software (Beckman Coulter).

Cell surface phenotyping: 100 μl of fresh EDTA blood was stained with three panels of antibodies including: (1) a modified Beckton Dickinson TBNK reagent Trucount tube to identify and determine the percentages and absolute counts of T, B and NK cells as well as T_reg_ cells; (2) a Beckman Coulter DURAClone IM T cell subset tube to assess maturation stages of T cells, covering naive, effector, memory and terminal differentiation stages; and (3) a modified Beckman Coulter DURAClone T_reg_ cell tube to assess FOXP3 T_reg_ cells and NK cell subsets. Details of the panels and indicative gating strategies are shown in Supplementary Table 7 and Supplementary Fig. 1. Tubes were processed according to the manufacturer’s instructions, acquired on a Navios flow cytometer and analyzed using Kaluza software.

Cytokine FluoroSpot: a million cryopreserved PBMCs were incubated in three wells of a freshly coated FluoroSpot plate (Mabtech) with 30 µg ml^−1^ of proinsulin (in-kind contribution from L. Vilela, Biomm, Brazil) or phosphate-buffered saline (as a negative control) for 48 h. IFNγ-, IL-17A- and IL-17F-secreting cells were detected according to the manufacturer’s instructions (Mabtech). Enumeration of spots was carried out using the IRIS FluoroSpot reader (Mabtech) and results presented as an SI (spot number in the presence of stimulus/spot number in the presence of appropriate negative control). All immune analyses were performed blinded to treatment group and then analyzed when a final locked dataset was sent to the PIs.

### Statistical analysis

#### Sample size considerations

The power calculation closely followed ref. ^71^ based on data for children and young adolescents aged 13–17 years as well as the T1DAL study in 12–35 year olds^72^. A sample size of 66 apportioned in a 2:1 ratio has a >85% power to detect a 0.2 nmol l^−1^ difference between the 2-h MMTT mean AUC values. C-peptide values of the intervention and placebo arms were assumed to be 0.5 and 0.3 (nmol l^−1^), respectively, at 12 months. It was planned for 72 participants (48 ustekinumab:24 placebo) to be recruited, allowing for approximately 10% lost to follow-up.

#### Randomization

Each randomization was via minimization incorporating a random element and incorporated two important prognostic factors: age (12–15 years versus 16–18 years) and screened peak C-peptide levels (0.2–0.7 nmol l^−1^ versus >0.7 nmol l^−1^) to ensure balance between treatment groups. Sealed Envelope Ltd (https://sealedenvelope.com/randomisation) supplied the minimization algorithm and randomization service and hosted the web-enabled allocation service.

#### Blinding

Participants, research staff and the trial office remained blinded, with only limited independent researchers at Swansea Trials Unit (STU) managing the code break list and any IMP-related queries from pharmacies.

#### Analysis population

All randomized participants who had not withdrawn from the study before the first day of treatment were included in trial analyses and analyzed according to the treatment allocated.

#### Analysis of primary outcome

The AUC was calculated using the trapezoidal method, not adjusted for baseline C-peptide but normalized for the 120-min period of the standard MMTT using the serum C-peptide value at each time point. Most C-peptide values fell between 0 and 1 and the distribution was positively skewed; they were transformed by log(1 + *x)* before treatment group comparisons. These comparisons were performed with an independent Student’s *t*-test at baseline. At weeks 28 and 52, treatment group differences were assessed with ANCOVA adjusting for the baseline C-peptide value, gender, age, HbA1c and exogenous insulin use. Results were back-transformed and summarized as the ratio of geometric means and percentage differences between groups^48,61^.

#### Analysis of secondary outcomes

Treatment group difference in secondary metabolic endpoints included HbA1c, daily insulin dose and IDAA1c. Treatment group differences at baseline were assessed with independent Student’s *t*-test. Week 12, 28 and 52 treatment group differences were analyzed with ANCOVA, adjusting for appropriate covariates. HbA1c and insulin use analyses post-baseline were adjusted by sex, age, HbA1c and insulin use at baseline. IDAA1c was calculated according to the formula: HbA1c (%) + (4 × insulin dose (units per kg per 24 h))^73^. Post-baseline IDAA1c analyses were adjusted by sex, age and IDAA1c at baseline. Results were summarized as differences in arithmetic means between groups.

#### Analysis of safety outcome

Safety assessments (that is, safety blood and urine tests and IMP-related adverse events during the course of the study) were counted in terms of both number of events and number of participants. For participants experiencing more than one adverse event, each participant was counted once at the highest level of severity for the event. No formal statistical testing was undertaken.

#### Data collection and analysis

Data were collected using electronic case report forms via MACRO 4.7. Data were analyzed using SPSS v.25 and STATA v.18.

#### Data visualization

Dot plots were constructed in R 4.3.0 using packages ggplot2, cowplot, scales and patchwork. All other plots were constructed in Stata.

### Reporting summary

Further information on research design is available in the Nature Portfolio Reporting Summary linked to this article.

## Online content

Any methods, additional references, Nature Portfolio reporting summaries, source data, extended data, supplementary information, acknowledgements, peer review information; details of author contributions and competing interests; and statements of data and code availability are available at 10.1038/s41591-024-03115-2.

## Supplementary information

Supplementary Information Supplementary Tables 1–7 and Fig. 1.

Reporting Summary

## Data Availability

Data are stored in the STU data repository (https://swanseatrialsunit.org). Data are to be shared with bona fide researchers who complete a data-sharing request form approved by a STU Data Sharing Committee. Individual patient data will be shared in datasets in a de-identified and anonymized format. All data-sharing requests should be made via STU@swansea.ac.uk. Initial enquiries for data sharing would receive a response within 5 working days. There would be an aim to release data within 28 working days, dependent on the completion of an appropriate Data Sharing Agreement.

## Code availability

Dot plots were constructed in R 4.3.0 using packages ggplot2, cowplot, scales and patchwork. All other plots were constructed in Stata.
