# Supplementary material for: Ustekinumab for type 1 diabetes in adolescents: a multicenter, double-blind, randomized phase 2 trial
Source: Nat Med. 2024 Jul 30;30(9):2657–66. doi: 10.1038/s41591-024-03115-2 (PMC11405276; doi:10.1038/s41591-024-03115-2)

nature medicine

Supplementary information https://doi.org/10.1038/s41591-024-03115-2

Ustekinumab for type 1 diabetes in adolescents: a multicenter, double-blind, randomized phase 2 trial

In the format provided by the authors and unedited

SUPPLEMENTARY MATERIALS

Supplementary Material Table 1. Geometric mean ratio (95% CI) of C-peptide AUC (nmol/L/minute) by treatment group.

Week	Ustekinumab (sd) n	Placebo (sd) n	Ustekinumab / placebo	95% Confidence Interval		p value* (two-sided)
				Lower	Upper	
-2	0.84 (0.28) n=47	0.87 (0.27) n=25	97%	74%	127%	0.82
28	0.49 (0.23) n=36	0.42 (0.22) n=19	115%	81%	163%	0.45
52	0.45 (0.20) n=41	0.3 (0.20) n=21	149%	108%	206%	0.02
*Assessed by Analysis of Coviarence (ANCOVA)						

Supplementary Material Table 2. Percentage of time hyperglycaemic at different thresholds across assessment timepoints (CGM data)

Timepoints of assessment	Placebo median (IQR) n	Ustekinumab median (IQR) n	p value* (two-sided)
**Percentage time > 7.8mmol/L**			
0 months	31.64 (22.87 - 50.14) n=19	35.95 (21.85 - 55.00) n=39	0.6
6 months	55.50 (33.09 - 68.17) n=19	52.10 (33.81 - 71.41) n=39	0.81
12 months	61.50 (47.48 - 74.56) n=17	55.62 (35.14 - 69.85) n=38	0.33
**Percentage time > 10mmol/L**			
0 months	12.25 (5.57 - 22.56) n=19	16.39 (5.00 - 30.42) n=39	0.66
6 months	29.06 (12.10 - 52.11) n=19	26.77 (14.31 - 45.63) n=39	0.65
12 months	37.89 (21.64 - 54.95) n=17	36.08 (15.85 - 47.06) n=38	0.5
**Percentage time > 13.9mmol/L**			
0 months	1.22 (0.60 - 4.65) n=19	2.36 (0.38 - 6.67) n=39	0.55
6 months	4.11 (1.90 - 25.58) n=19	7.13 (0.98 - 16.36) n=39	0.65
12 months	12.11 (3.67 - 28.15) n=17	8.69 (2.25 - 20.87) n=38	0.39
* assessed by Mann-Whitney U tests			

Supplementary Material Table 3. Percentage of time participants were hypoglycaemic (CGM data)

Timepoints of assessment	Control median (IQR) n	Ustekinumab median (IQR) n	p value* (two-sided)
**Percentage time < 3.9mmol/L**			
0 months	2.94 (1.22 - 10.16) n=19	3.14 (0.79 - 6.39) n=39	0.67
6 months	2.64 (0.83 - 3.76) n=19	2.05 (0.65 - 5.42) n=39	0.88
12 months	1.10 (0.52 - 3.14) n=17	1.80 (0.44 - 5.53) n=38	0.27
**Percentage time < 3mmol/L**			
0 months	0.18 (0.00 -0.85) n=19	0.12 (0.00 - 1.09) n=39	0.99
6 months	0.18 (0.00 - 0.44) n=19	0.15 (0.00 - 0.99) n=39	0.84
12 months	0.03 (0.00 - 0.33) n=17	0.17 (0.00 - 0.53) n=38	0.66
* assessed by Mann-Whitney U tests			

Supplementary Table 4a. Distribution of clinical hypoglycaemic events by participants by groups

No of hypos (n=2946)	No. of participants			
	Control (n=25)		Ustekinumab (n=47)	
	n	%	n	%
0	1	4	3	6
1-9	2	8	7	15
10-19	6	24	6	13
20-29	1	4	6	13
30-39	1	4	6	13
40-49	6	24	4	9
50-59	1	4	5	11
60-69	2	8	2	4
70-79	2	8	2	4
80-89	0	0	1	2
90-99	1	4	0	0
100+	2	8	5	11

Supplementary Table 4b. Levels of severity of hypoglycaemic events assessed by clinicians by groups

Level of Hypo	Incidence per person year		Incidence Rate Ratio (IRR)	95% CI for IRR	p value* (two-sided)
	Control (n=22)	Ustekinumab (n=44)	Control / Ustekinumab		
Level 1 – A glucose alert value of > 3.0 but ≤ 3.9 mmol/L (n=2228)	34.88	31.61	1.1	0.71, 1.72	0.67
Level 2 – A glucose level of ≤ 3.0 mmol/L – clinically important hypoglycaemia (n=615)	8.13	9.55	0.85	0.51, 1.42	0.54
Level 3 – Severe hypoglycaemia, as defined by the ADA denotes severe cognitive impairment requiring external assistance for recovery (n=1)	1 person (407) in the control group had 1 hypo assessed to be at Level 3				
Not classified (n=65)	2.12	0.32	6.68	1.21, 36.82	0.03 [a]
*assessed by negative binominal test					

Supplementary Table 4c. Clinical characterisation of hypoglycaemic events assessed by clinicians by groups

Clinical characterisation of Hypoglycaemic event	Incidence per person year		Incidence Rate Ratio (IRR)	95% CI for IRR	p value* (two-sided)
	Control (n=22)	Ustekinumab (n=44)	Control / Ustekinumab		
Severe hypoglycaemia (n=10)	0.13	0.16	0.79	0.19, 3.32	0.74
Documented symptomatic hypoglycaemia (n=2665)	39.54	39	1.01	0.66, 1.57	0.95
Probable symptomatic hypoglycaemia (n=169)	4.95	1.13	4.36	1.39, 3.69	0.012 [a]
Hypoglycaemia unlikely (n=59)	0.33	1.16	0.29	0.04, 2.09	0.29
Reported as SAE	0	0			
*assessed by negative binominal test					

Supplementary Table 5a. Differences in mean participant reported outcomes by groups at 6 and 12 months, adjusted by baseline

**Participant reported PROMS**	**Visit**	**Control (sd) n**	**Ustekinumab (sd) n**	**Ustekinumab - Control**	**95% CI Lower**	**95% CI Upper**	**p value* (two-sided)**	**Adj R^2**
PedsQL (0-100)<br><br>*Higher score, better generic HRQoL*	Week -2 N=67	84.87 (11.31) n=24	76.52 (13.08) n=43	-8.36	-14.47	-2.24	0.008*	NA
	Week 28 N=60	79.19 (10.51) n=21	80.72 (10.30) n=39	1.54	-4.29	7.36	0.6	0.3
	Week 52 N=58	80.78 (8.89) n=21	80.12 (8.72) n=37	-0.662	-5.65	4.33	0.79	0.43
PedsQL diabetes (0-100)<br><br>*Higher score, better diabetes-specific HRQoL*	Week -2 N=70	76.81 (13.00) n=25	75.61 (12.58) n=45	-1.2	-7.64	5.24	0.71	NA
	Week 28 N=63	75.48 (8.94) n=22	73.87 (8.93) n=41	-1.61	-6.35	3.12	0.5	0.48
	Week 52 N=64	74.48 (10.87) n=22	73.00 (10.87) n=42	-1.48	-7.2	4.25	0.61	0.45
DTSQ (0-36)<br><br>*The higher, the more satisfied*	Week-2 N=66	24.61 (4.04) n=23	24.81 (3.84) n=43	0.25	-1.86	2.28	0.84	NA
	Week 28 N=56	23.82 (4.61) n=18	24.11 (4.60) n=38	0.292	-2.35	2.94	0.83	0.05
	Week 52 N=58	23.54 (4.24) n=19	24.92 (4.24) n=39	1.38	-1.00	3.76	0.25	0.14
HypoFear-Behaviour (0-4)<br><br>*Higher score, greater tendency to avoid hypo or its negative consequences*	Week-2 N=67	1.7 (0.80) n=24	1.81 (0.80) n=43	0.11	-0.3	0.52	0.59	NA
	Week 28 N=60	1.73 (0.54) n=21	1.77 (0.54) n=39	0.04	-0.26	0.33	0.81	0.33
	Week 52 N=61	1.81 (0.49) n=21	1.82 (0.49) n=40	0.01	-0.25	0.28	0.92	0.36
HypoFear-Worry (0-4)<br><br>*Higher score, more worry concerning hypo and its consequences*	Week-2 N=69	0.77 (0.54) n=25	0.91 (0.79) n=44	0.14	-0.18	0.46	0.38	NA
	Week 28 N=62	0.88 (0.47) n=22	0.94 (0.25) n=40	0.06	-0.19	0.32	0.61	0.45
	Week 52 N=63	0.86 (0.49) n=22	0.96 (0.49) n=41	0.1	-0.16	0.36	0.46	0.25

HypoFear-Total (0-4)<br><br>*Higher score, greater fear of hypo*	Week -2 N=70	1.09 (0.47) n=25	1.21 (0.64) n=45	0.12	-0.17	0.42	0.36	NA
	Week 28 N=63	1.23 (0.39) n=22	1.25 (0.40) n=41	0.11	-0.19	0.23	0.84	0.45
	Week 52 N=64	1.24 (0.42) n=22	1.29 (0.42) n=42	0.05	-0.17	0.27	0.65	0.32
*assessed by Analysis of Covariance (ANCOVA)								

Supplementary Table 5b. Differences in mean parent reported outcomes by groups at 6 and 12 months, adjusted by baseline

Parent reported PROMS	Visit	Control (sd) n	Ustekinumab (sd) n	Ustekinumab - control	95% CI Lower	Upper	p value* (two-sided)	Adj R^2
PedsQL (0-100)	Week -2 N=67	79.07 (16.08) n=22	78.28 (16.95) n=45	-0.79	-9.37	7.8	0.85	NA
Higher score, better generic HRQoL	Week 28 N=59	76.44 (13.20) n=21	80.86 (13.19) n=38	4.42	-2.76	11.6	0.22	0.21
	Week 52 N=59	80.62 (13.03) n=19	79.58 (13.03) n=40	-1.04	-8.33	6.25	0.78	0.26
PedsQL diabetes (0-100)	Week -2 N=68	67.54 (15.63) n=22	71.42 (15.48) n=46	4.88	-4.28	12.04	0.34	NA
Higher score, better diabetes-specific HRQoL	Week 28 N=59	77.75 (13.75) n=21	80.13 (13.75) n=38	2.38	-5.13	9.89	0.53	0.15
	Week 52 N=59	82.37 (13.29) n=19	78.74 (13.28) n=40	-3.63	-11.07	3.81	0.33	0.23
DTSQ (0-36)	Week-2 N=60	23.53 (5.90) n=19	26.32 (4.50) n=41	2.79	-0.33	5.92	0.08	NA
The higher, the more satisfied	Week 28 N=49	22.83 (4.03) n=18	25.00 (4.01) n=31	2.17	-0.26	4.6	0.08	0.25
	Week 52 N=49	23.70 (4.48) n=16	24.82 (4.42) n=33	1.12	-1.65	3.89	0.42	0.18
HypoFear-Behaviour (0-4)	Week-2 N=67	1.92 (0.70) n=22	1.74 (0.88) n=45	-0.18	-0.58	0.22	0.38	NA
Higher score, greater tendency to avoid hypo or its negative consequences	Week 28 N=60	1.53 (0.55) n=21	1.53 (0.56) n=39	0	-0.3	0.3	1	0.13
	Week 52 N=59	1.65 (0.44) n=19	1.59 (0.44) n=40	0.06	-0.31	0.18	0.61	0.43

HypoFear-Worry (0-4)<br><br>*Higher score, more worry concerning hypo and its consequences*	Week-2 N=67	1.54 (0.77) n=22	1.38 (0.91) n=45	-0.16	-0.59	0.27	0.45	NA
	Week 28 N=59	1.49 (0.82) n=21	1.42 (0.81) n=38	-0.07	-0.51	0.38	0.77	0.22
	Week 52 N=59	1.11 (0.65) n=19	1.37 (0.67) n=40	0.26	-0.12	0.64	0.17	0.26
HypoFear-Total (0-4)<br><br>*Higher score, greater fear of hypo*	Week - 2 N=67	1.69 (0.67) n=22	1.53 (0.78) n=45	-0.17	-0.54	0.2	0.37	NA
	Week 28 N=60	1.50 (0.64) n=21	1.47 (0.62) n=39	-0.025	-0.37	0.32	0.88	0.22
	Week 52 N=59	1.31 (0.53) n=19	1.45 (0.53) n=40	0.13	-0.16	0.43	0.37	0.36
*assessed by Analysis of Covariance (ANCOVA)								

Supplmenentary Material Table 6a. Adverse Events severity by treatment group.

Severity	Placebo (n=25)		Ustekinumab (n=47)	
	No. of events	No. of participants (%)	No. of events	No. of participants (%)
Mild	113	22 (88%)	232	41 (87%)
Moderate	12	8 (32%)	21	15 (32%)
Severe	0	0	0	0
Total	125	22 (88%)	253	41 (87%)

Supplementarary Material Table 6b. Frequency of Adverse Evenets catagorised in System Ogan Class by Treatment group and Severity

AE severity	System Organ Class	Saline	Ustekinumab	Total
Mild	Infections and infestations	33	74	107
	Immune system disorders	7	15	22
	Nervous system disorders	24	57	81
	Respiratory, thoracic and mediastinal disorders	2	0	2
	Gastrointestinaldisorders	20	25	45
	Skin and subcutaneous tissue disorders	8	12	20
	Musculoskeletal and connective tissue disorders	6	6	12
	General disorders and administration site conditions	10	20	30
	Others	3	23	26
		113	232	345
Moderate	Infections and infestations	2	6	8
	Immune system disorders	0	1	1
	Nervous system disorders	2	3	5
	Respiratory, thoracic and mediastinal disorders	0	1	1
	Gastrointestinaldisorders	2	4	6
	Skin and subcutaneous tissue disorders	1	0	1
	Musculoskeletal and connective tissue disorders	2	3	5
	General disorders and administration site conditions	3	2	5
	Others	0	1	1

Supplementarary Material Table 6c. Frequency of Adverse effects in free text description by Treatment group and Severity

**MILD**				
System Organ Class	Description of AE	Saline	Ustekinumab	Total
Infections and infestations	Bacterial infection	0	1	1
	Candidal Vaginitis Thrush	1	0	1
	Chest infection, cough, runny nose, fever	1	0	1
	Chesty Cough	1	0	1
	cold	0	4	4
	Cold	1	5	6
	COLD	2	1	3
	Cold (URTI)	0	4	4
	Cold and sore throat	0	1	1
	Cold Sore	0	1	1
	Cold sore on side of mouth	0	1	1
	Cold symptoms	0	2	2
	Cold Symptoms	0	1	1
	Common cold	1	0	1
	Conjunctivitus	1	0	1
	Coryzal symptoms	1	0	1
	cough	2	0	2
	Cough	3	1	4
	Cough + cold symptoms	1	0	1
	Cough, coryzal + runny nose	1	0	1
	COVID	0	1	1
	COVID 19	0	1	1
	COVID infection	0	1	1
	Covid-19	0	2	2
	COVID-19	0	1	1
	COVID-19 infection	0	2	2
	COVID-19 POSITIVE	1	0	1
	Ear infection	0	1	1
	ear infection	0	1	1
	Ear Infection	0	1	1
	Febrile Viral illness, cough	1	0	1
	fever	1	0	1
	Fever	0	1	1
	Flu like symptoms	0	1	1
	Flu symptoms	1	1	2
	Head cold	1	0	1
	Headcold	0	1	1
	HEADCOLD	0	1	1
	Headcold (headaches, sore throat etc)	0	1	1
	Infected insect bite	0	1	1
	Infection of ear piercing	1	0	1

	Lower Respiratory Tract Infection	0	1	1
	LRTI	0	1	1
	Nits and headlice	0	1	1
	Pharyngitis	0	1	1
	runny nose	1	0	1
	Runny Nose	0	1	1
	Scarlett Fever	0	1	1
	Sore throat	0	1	1
	Sore Throat	1	5	6
	sore throat general aches before Christmas not sure exact dates 20/12/19 - 23/12/19	1	0	1
	Sore throat, flu	1	0	1
	Sore throat, temperature, nausea, chesty cough	1	0	1
	Sore throat/headache	0	1	1
	SYMPTOMS OF COLD AND COUGH	1	0	1
	Tickly cough and bad cold	1	0	1
	Tonsilitis	1	0	1
	tooth infection	0	1	1
	Upper respiratory tract infection	0	2	2
	Upper Respiratory Tract Infection	0	4	4
	Upper respiratory tract infection (common cold)	0	1	1
	Upper Respiratory Tract infection (common cold)	0	3	3
	Upper Respiratory Tract Infection (common cold)	0	3	3
	UTI	1	0	1
	Viral Illness	1	0	1
	Viral illness - cold	0	1	1
	Viral illness- cold	0	1	1
	Viral illness, headache and nausea	1	0	1
	Viral infection	0	1	1
	Viral respiratory infection	0	1	1
	Viral URTI	0	2	2
	virus/mild illness with myalgia	0	1	1
	Total	33	74	107
Immune system disorders	Allergic Reaction To Cat	1	0	1
	Allergies	1	8	9
	Enlarged lymph nodes on right side	0	1	1
	Fever after COVID vaccination	0	1	1
	Hay fever	0	1	1
	Hayfever	2	2	4
	High Eosinophils levels	0	1	1
	Lymphadenitis left auxilla	1	0	1
	Supraclavical lymph node, small	1	0	1
	Tiredness after COVID vaccination	0	1	1
	urticaria	1	0	1

	Total	7	15	22
Nervous system disorders	Dizzy	0	2	2
	Faint	0	1	1
	Head ache, tummy ache	1	0	1
	headache	6	4	10
	Headache	10	32	42
	HEADACHE	1	0	1
	Headache and nausea	1	0	1
	headache exact date in november not known 24hrs	1	0	1
	Headache/feeling unwell following Screen 1 blood samples	0	1	1
	Headaches	2	3	5
	Headaches, no start date & no pattern but has been since starting the trial. Reported 27/8/19 at TX2	1	0	1
	Heasdache	0	1	1
	Worsening Headaches	0	1	1
	migraine	1	0	1
	Migraine	0	12	12
	Total	24	57	81
Respiratory, thoracic and mediastinal disorders	breathlessness	1		1
	Heart palpitations	1		1
		2		2
Gastrointestinaldisorders	abdo pain	0	1	1
	Abdominal Cramps	1	0	1
	Abdominal Crapms	2	0	2
	Abdominal Pain	0	1	1
	Abdominal pain - stomach ulcer	0	1	1
	Abdominal pain loose stools nausea vomiting	0	1	1
	Abdominal Pain loose stools nausea vomiting	0	1	1
	Diarrhoea	3	3	6
	Intermittent Diarrhoea	0	1	1
	Loose Stools	0	1	1
	Dizziness (car sickness)	1	0	1
	Gastroenteritis	0	1	1
	Haemarrhoids	0	1	1
	Increase Appetite	1	0	1
	loss of appetite - dates approximate	1	0	1
	Mild Gastroenteritis	1	0	1
	Nausea	1	1	2
	nausea (car sickness)	1	0	1
	Nauseous	0	2	2
	Norovirus	0	1	1
	Pain in tummy	1	0	1
	stomach cramp	1	0	1

	stomach cramps	1	0	1
	Stomach pain	0	1	1
	Stomach/back ache	1	0	1
	tummy ache	0	1	1
	Tummy ache	1	0	1
	Tummy pain	1	0	1
	vomiting	1	1	2
	Vomiting	0	1	1
	VOMITING	0	1	1
	Vomiting + raised ketones (0.7)	0	1	1
	Vomiting and headache	0	1	1
	vomiting, headache	1	0	1
	Vomiting/Nausea	0	1	1
	Vomitted after esure drink during V5	0	1	1
	Total	20	25	45
Skin and subcutaneous tissue disorders	Acne	0	2	2
	Acne (face and shoulders)	1	0	1
	Adverse reaction to acne gel (adapalene 0.1 and benzoyl peroxide 2.5 - Epiduo)	1	0	1
	Eczema	0	1	1
	Eczematous skin lesion 2nd and 3rd left toes	0	1	1
	hairloss	1	0	1
	Heat Rash	0	1	1
	Insect bite	0	1	1
	Itchy scalp	1	0	1
	Lipohypertrophy at abdomen	1	0	1
	non blanching rash on both legs	0	1	1
	non blanching rash to shoulders, arms, torso and legs	0	1	1
	Rash	1	0	1
	Rash (back of neck & abdomen)	0	1	1
	RASH (FRONT OF EARS AND NECK)	0	1	1
	Rash on face	0	1	1
	Rash over hands	1	0	1
	right arm erythema	1	0	1
	Stretch Marks	0	1	1
	Total	8	12	20
Musculoskeletal and connective tissue disorders	back pain	1	0	1
	Foot Blisters	0	1	1
	Groin Pain	0	1	1
	Hip Injury	1	0	1
	Injured finger	0	1	1
	Injury to right little finger	0	1	1
	Insulin related Ankle Oedema	0	1	1
	Knee pain	0	1	1
	PAIN BOTH LEGS	1	0	1

	Pain in extremity	1	0	1
	Planter Fasciitis	1	0	1
	shoulder pain	1	0	1
	Total	6	6	12
General disorders and administration site conditions	abnormal albumin/creatinine ratio in urine	0	1	1
	Albuminuria	0	1	1
	Attended A & E for High blood sugars	1	0	1
	blood in urine	0	1	1
	Delayed period	0	1	1
	Electric shock	1	0	1
	Goitre, found during ultrasound investigations for enlarge lymph node on 18/9/19. Referred to ENT	0	1	1
	Headlice infestation	0	1	1
	Hypocalcemia	0	1	1
	Injection site erythema	0	1	1
	Low Iron	0	1	1
	Low magnesium	1	0	1
	Microalbuminuria	0	1	1
	Nosebleeds	1	0	1
	Pain at injection site	0	1	1
	pain during injection procedure	0	1	1
	Pain from libre device	1	0	1
	Pain from tongue piercing	1	0	1
	Proteinuria	0	1	1
	Raised HbA1c level	0	1	1
	Raised Urine albumin/creatinine ratio 35.6 mg/mmol	1	0	1
	redness and itching at injection site immediately after study drug given	0	1	1
	Slight fullness at insulin injection site	1	0	1
	Small red bump on injection site (not itchy, not painful)	0	1	1
	tiredness	0	1	1
	total cholesterol and LDLC out of range	0	1	1
	vaginal dryness	1	0	1
	Vitamin D deficiency	0	1	1
	weight loss	1	0	1
	weight loss noted	0	1	1
	Total	10	20	30
Others	Anaemia	0	1	1
	Dental filling	0	1	1
	Dental Pain	0	2	2
	Dental Pain following dental surgery	0	1	1
	Dental surgery	0	1	1
	Dental Surgery	0	1	1
	Dental work/pain	0	1	1

	Fatigue	1	0	1
	intermittent epistaxis	0	1	1
	Low haemoglobin/ anaemia	1	0	1
	Period	0	1	1
	Period pain	0	1	1
	Period Pain	0	2	2
	Period pains	0	1	1
	protein in urine	0	1	1
	Sore mouth (lower teeth braces fitted)	0	1	1
	Sore mouth (ulcers from braces)	0	2	2
	Teeth extractions x2	0	2	2
	Tooth Pain	1	0	1
	Toothache	0	2	2
	Toothpain (brace)	0	1	1
	Total	3	23	26
**MODERATE**				
System Organ Class	Description of AE	Saline	Ustekinumab	Total
Infections and infestations	COVID-19	1	0	1
	fever	0	1	1
	Flu/cold symptoms, headache, nausea, aching	1	0	1
	Sore Throat after Tonsillectomy	0	1	1
	Tonsillectomy	0	1	1
	tonsillitis	0	1	1
	Unwell, high temp for 24hrs. COVID neg	0	1	1
	upper respiratory tract infection	0	1	1
	Total	2	6	8
Immune system disorders	Hay fever		1	1
	Total		1	1
Nervous system disorders	CT Head following a fall	1	0	1
	Headache	0	1	1
	Headache and Abdominal Pain	0	1	1
	Non-Glycaemic Fit	0	1	1
	vasovagal episode	1	0	1
	Total	2	3	5
Respiratory, thoracic and mediastinal disorders	Suspected Pulminary Embolism		1	1
	Total		1	1
Gastrointestinaldisorders	Abdo pain / colic	1	0	1
	Abdominal Cramps	1	0	1
	Abdominal pain	0	1	1
	Lower abdomen pain/cramps	0	1	1
	Nausea & vomiting	0	1	1
	Vomitting	0	1	1
	Total	2	4	6
Skin and subcutaneous tissue disorders	Skin Rash	1		1

		1		1
Musculoskeletal and connective tissue disorders	4 broken toes on left foot	0	1	1
	broken left thumb	1	0	1
	Excision of inflamed ingron toenail. L	0	1	1
	Nasal fracture, bleeding, manipulation to re-allign required	1	0	1
	Numbness to left arm	0	1	1
	Total	2	3	5
General disorders and administration site conditions	Diagnosis of Diabetic Maculopathy	1	0	1
	High TSH level	0	1	1
	HYPOTHYROIDISM	0	1	1
	Raised urine ACR Albumin/Creatinine Ratio 67.7 mg/mmol	1	0	1
	Raised Urine Albumin/creatinine ratio 73.6 mg/mmol	1	0	1
	Total	3	2	5
Others	Anaemia		1	1
	Total		1	1

Supplementary Material Table 7: Details of antibody panels used in flow cytometry

Intracellular cytokine					
**Marker**	**Antibody clone**	**Fluorochrome**	**Manufacturer**	**Format**	**Dilution**
IFN-g	45.15	FITC	Beckman Coulter	Duraclone	n/a
GM-CSF	BVD2-21C11	PE-dazzle 594	BioLegend	Drop-in	1:50
CD8	B9.11	PC5.5	Beckman Coulter	Drop-in	1:50
IL-4	MP4-25D2	PC7	Beckman Coulter	Duraclone	n/a
CD4	13B8.2	APC	Beckman Coulter	Duraclone	n/a
IL-2	MQ1-17H12	APC-R700	Beckton Dickinson	Drop-in	1:50
CD3	UCHT1	APC-A750	Beckman Coulter	Duraclone	n/a
IL-17A	BL168	PacBlue	Beckman Coulter	Duraclone	n/a
Live-dead	n/a	Live-dead yellow	Invitrogen	Drop-in	1:50
T cell subset					
**Marker**	**Antibody clone**	**Fluorochrome**	**Manufacturer**	**Format**	**Dilution**
CD45RA	2H4	FITC	Beckman Coulter	Duraclone	n/a
CCR7	G043H7	PE	Beckman Coulter	Duraclone	n/a
CD28	CD28.2	ECD	Beckman Coulter	Duraclone	n/a
PD1	PD1.3.5	PC5.5	Beckman Coulter	Duraclone	n/a
CD27	1A4.CD27	PC7	Beckman Coulter	Duraclone	n/a
CD4	13B8.2	APC	Beckman Coulter	Duraclone	n/a
CD8	B9.11	APC-A700	Beckman Coulter	Duraclone	n/a
CD3	UCHT-1	APC-A750	Beckman Coulter	Duraclone	n/a
CD57	NC1	PacBlue	Beckman Coulter	Duraclone	n/a
CD45	J33	KrOrange	Beckman Coulter	Duraclone	n/a
TBNK/Treg count (lyse no wash)					
**Marker**	**Antibody clone**	**Fluorochrome**	**Manufacturer**	**Format**	**Dilution**
CD3	SK7	FITC	Beckton Dickinson	Cocktail	1:20
CD16	B73.1	PE	Beckton Dickinson	Cocktail	1:20
CD56	NCAM16.2	PE	Beckton Dickinson	Cocktail	1:20
CD127	A019D5	PE-dazzle 594	BioLegend	Drop-in	1:100
CD45	2D1	PerCP-Cy5.5	Beckton Dickinson	Cocktail	1:20
CD4	SK3	PE-Cy7	Beckton Dickinson	Cocktail	1:20
CD19	SJ25C1	APC	Beckton Dickinson	Cocktail	1:20
CD8	SK1	APC-Cy7	Beckton Dickinson	Cocktail	1:20
CD25	2A3	BV421	Beckton Dickinson	Drop-in	1:100
CD25	M-A251	BV421	Biolegend	Drop-in	1:100
Treg + NK subsets					
**Marker**	**Antibody clone**	**Fluorochrome**	**Manufacturer**	**Format**	**Dilution**
CD45RA	2H4LDH11LDB9(2H4)	FITC	Beckman Coulter	Duraclone	n/a
CD25	B1.49.9	PE	Beckman Coulter	Duraclone	n/a
CD25	M-A251	PE	Beckton Dickinson	Drop in	1:40
CD56	B159	PE-CF594	Beckton Dickinson	Drop in	1:200
CD39	BA54	PC5.5	Beckman Coulter	Duraclone	n/a
CD4	SFCI12T4D11(T4)	PC7	Beckman Coulter	Duraclone	n/a
FOXP3	259D	AF647	Beckman Coulter	Duraclone	n/a
CD16	3G8	AF700	Beckton Dickinson	Drop in	1:200
CD3	UCHT-1	APC-A750	Beckman Coulter	Duraclone	n/a
Helios	22F6	PacBlue	Beckman Coulter	Duraclone	n/a
CD45	J33	KrOrange	Beckman Coulter	Duraclone	n/a

Supplementary Material Figure 1:
Gating schemes for antibody panels used in flow cytometry

TBNK/Treg count

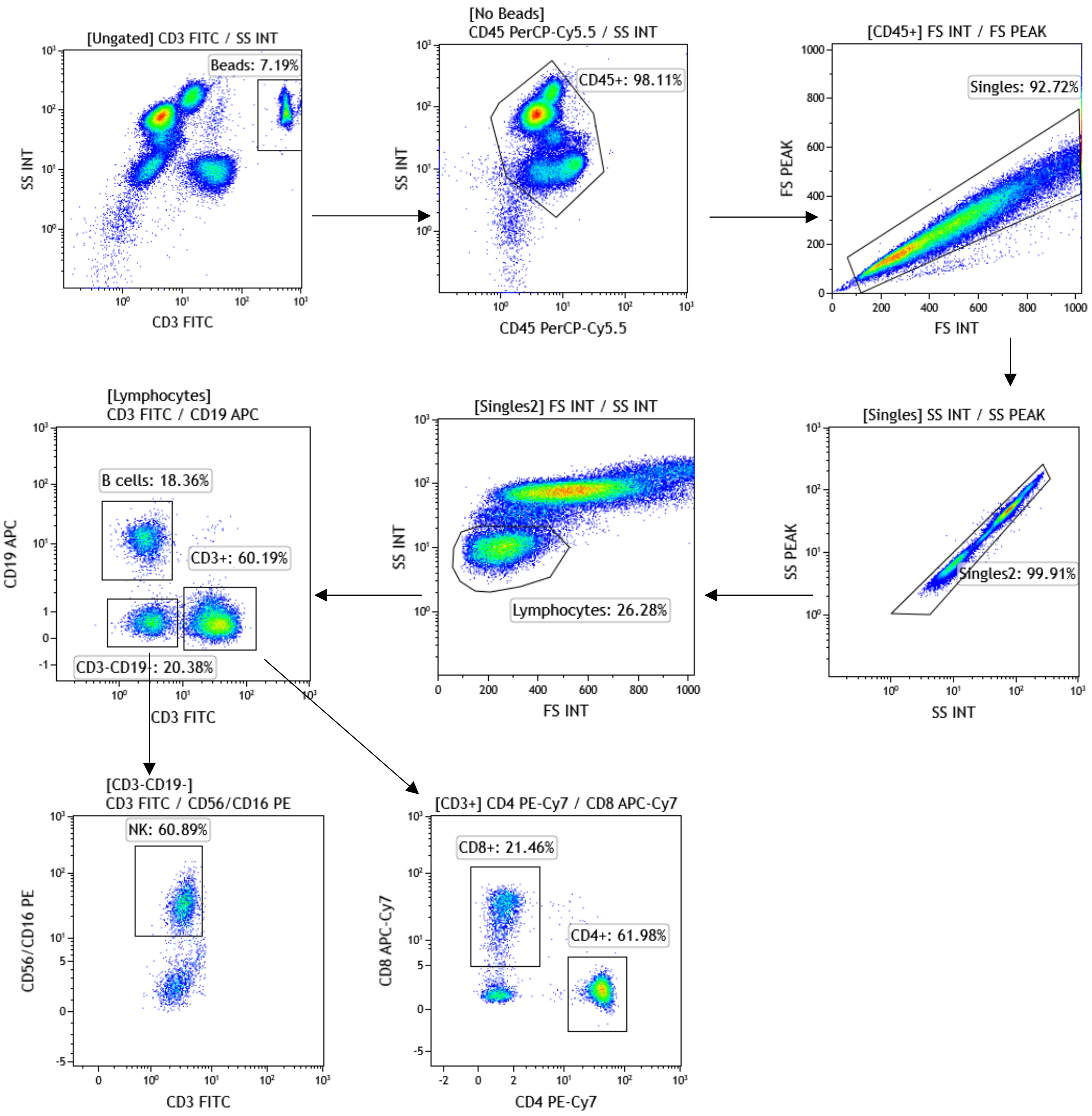

T-cell subset panel

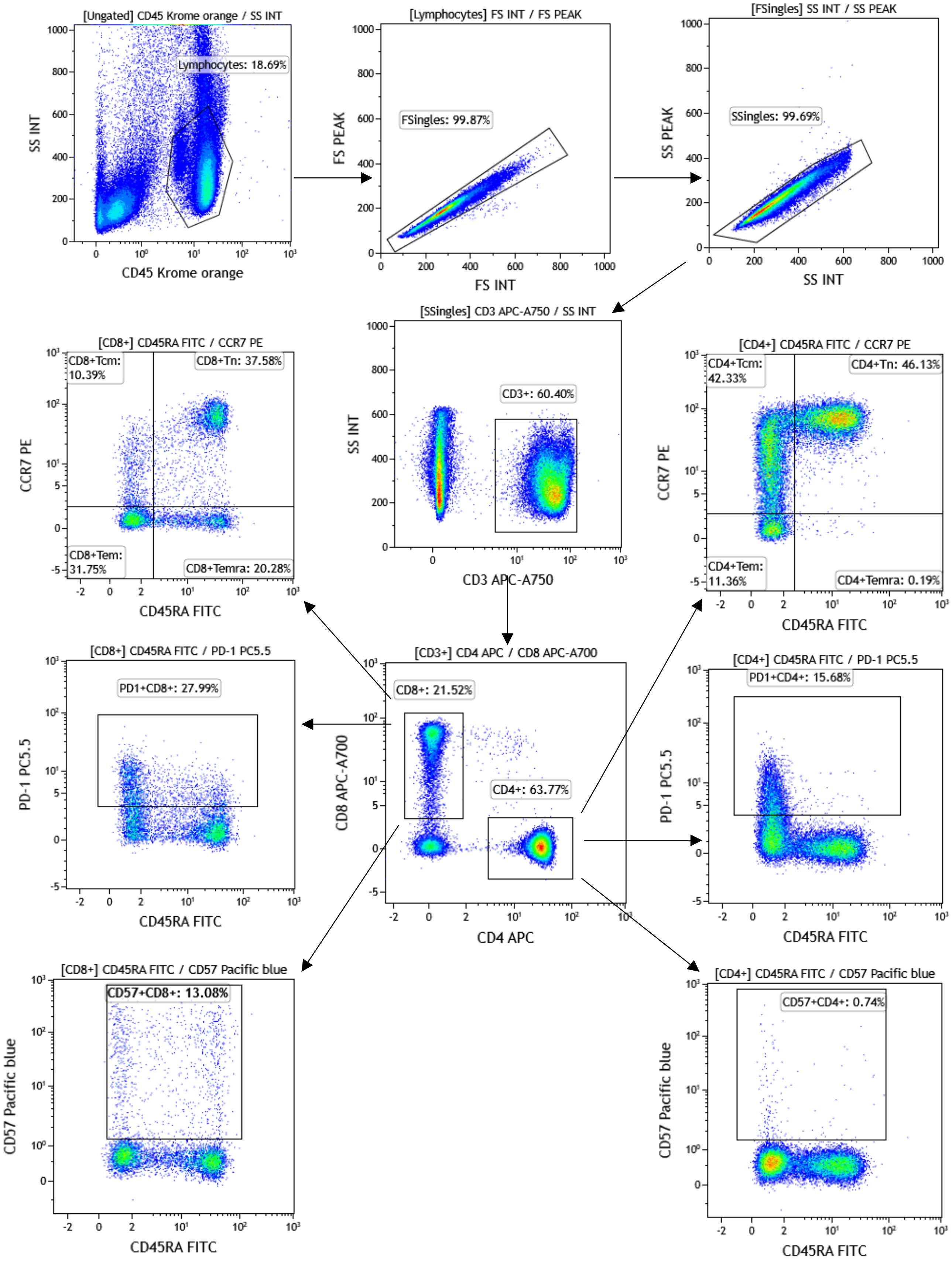

Treg + NK subsets panel

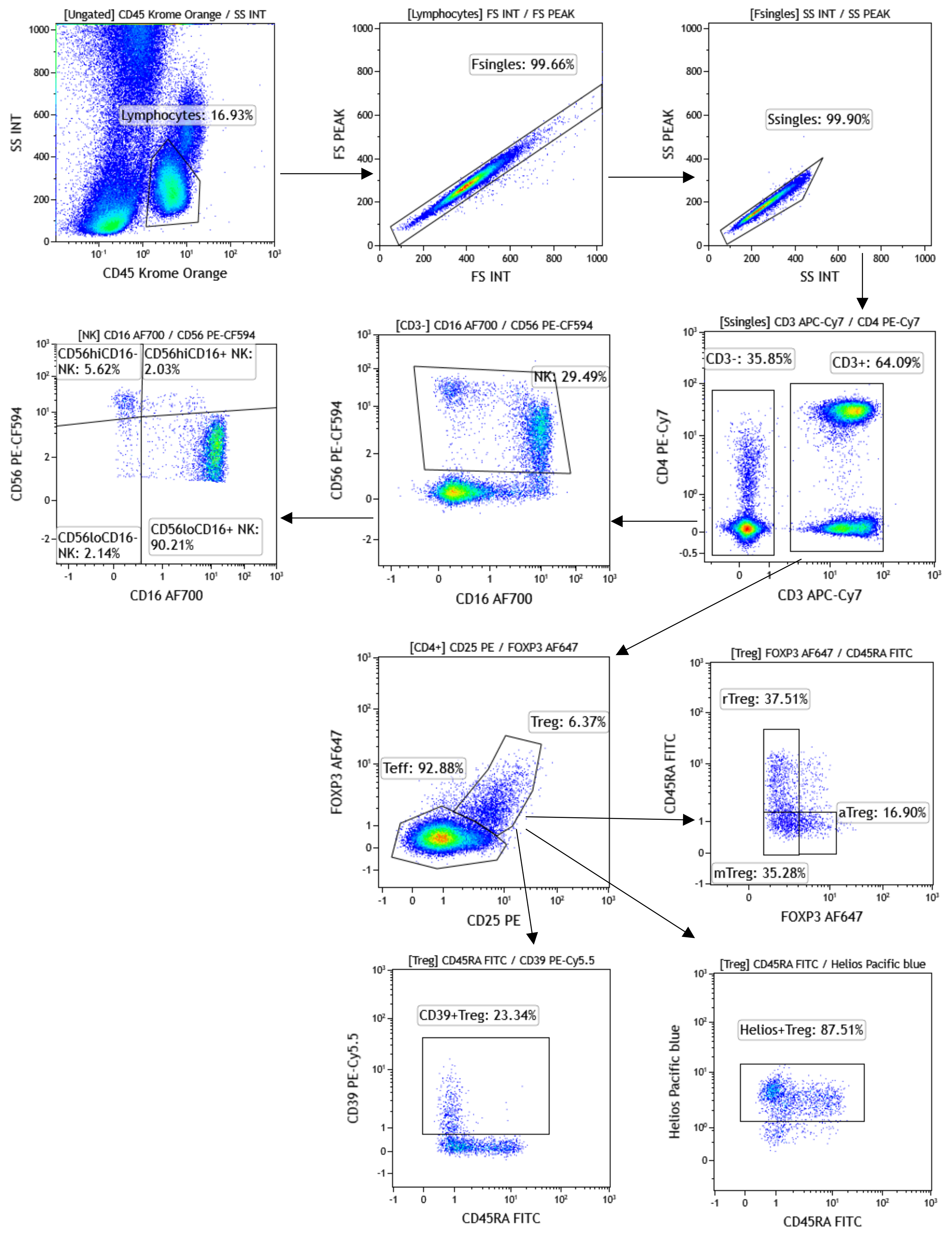

Intracellular cytokine panel

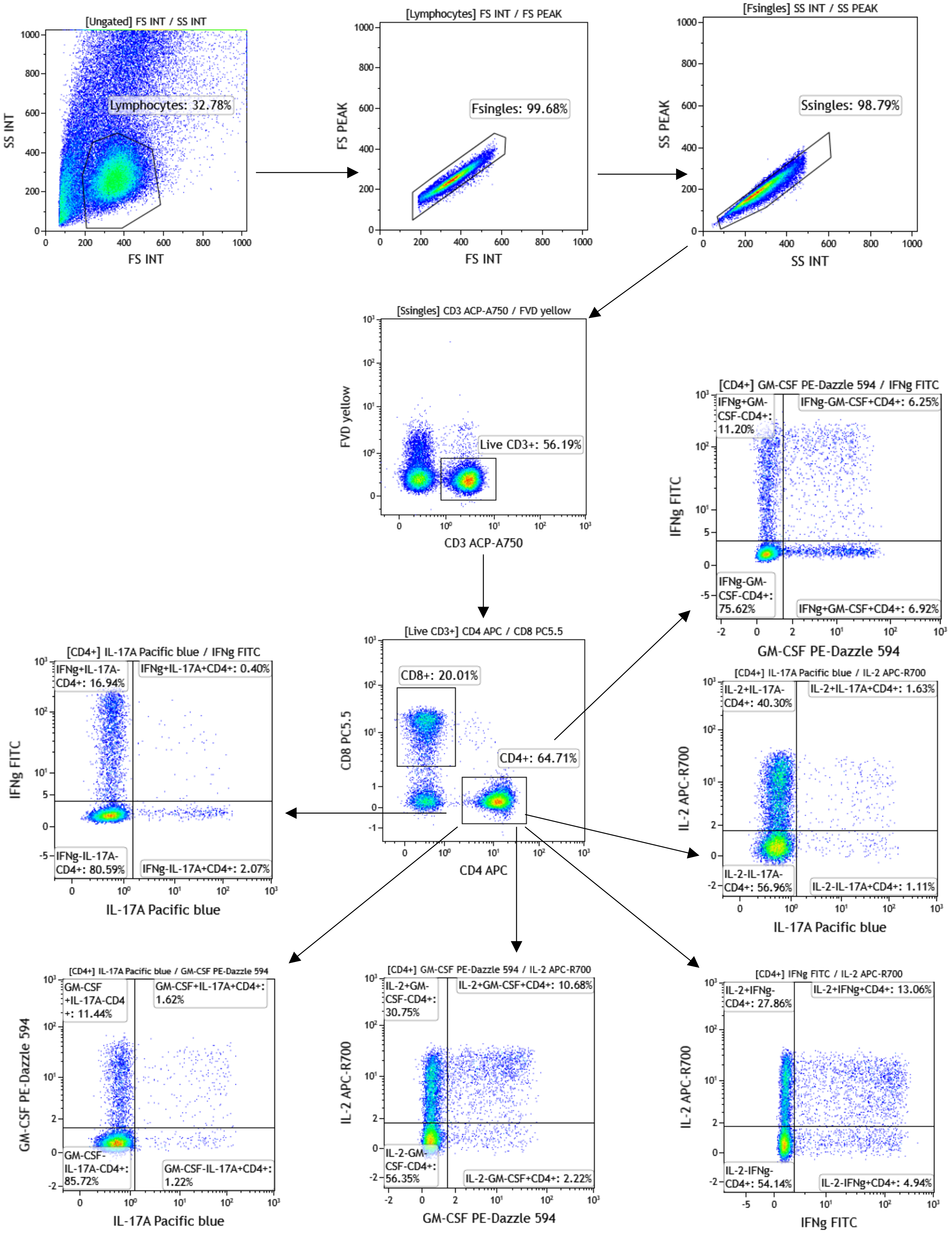

Supplement: Supplementary file 1 — Supplementary Tables 1–7 and Fig. 1. [file 41591_2024_3115_MOESM1_ESM.pdf]
