# Supplementary material for: Ustekinumab for type 1 diabetes in adolescents: a multicenter, double-blind, randomized phase 2 trial
Source: Nat Med. 2024 Jul 30;30(9):2657–66. doi: 10.1038/s41591-024-03115-2 (PMC11405276; doi:10.1038/s41591-024-03115-2)
Supplement: Supplementary file 2 — Reporting Summary [file 41591_2024_3115_MOESM2_ESM.pdf]

nature portfolio

Corresponding author(s): Dr Danijela Tatovic

Last updated by author(s): May 25, 2024



# Reporting Summary

Nature Portfolio wishes to improve the reproducibility of the work that we publish. This form provides structure for consistency and transparency in reporting. For further information on Nature Portfolio policies, see our Editorial Policies and the Editorial Policy Checklist.

## Statistics

For all statistical analyses, confirm that the following items are present in the figure legend, table legend, main text, or Methods section.

| n/a | Confirmed | |
|---|---|---|
| ☐ | ☒ | The exact sample size (*n*) for each experimental group/condition, given as a discrete number and unit of measurement |
| ☒ | ☐ | A statement on whether measurements were taken from distinct samples or whether the same sample was measured repeatedly |
| ☐ | ☒ | The statistical test(s) used AND whether they are one- or two-sided<br>*Only common tests should be described solely by name; describe more complex techniques in the Methods section.* |
| ☐ | ☒ | A description of all covariates tested |
| ☐ | ☒ | A description of any assumptions or corrections, such as tests of normality and adjustment for multiple comparisons |
| ☐ | ☒ | A full description of the statistical parameters including central tendency (e.g. means) or other basic estimates (e.g. regression coefficient) AND variation (e.g. standard deviation) or associated estimates of uncertainty (e.g. confidence intervals) |
| ☐ | ☒ | For null hypothesis testing, the test statistic (e.g. *F*, *t*, *r*) with confidence intervals, effect sizes, degrees of freedom and *P* value noted<br>*Give P values as exact values whenever suitable.* |
| ☒ | ☐ | For Bayesian analysis, information on the choice of priors and Markov chain Monte Carlo settings |
| ☒ | ☐ | For hierarchical and complex designs, identification of the appropriate level for tests and full reporting of outcomes |
| ☐ | ☒ | Estimates of effect sizes (e.g. Cohen's *d*, Pearson's *r*), indicating how they were calculated |

*Our web collection on statistics for biologists contains articles on many of the points above.*

## Software and code

Policy information about availability of computer code

| | |
|---|---|
| Data collection | electronic Case report Forms via MACRO 4.7 |
| Data analysis | SPSS version 25; STATA version 18; Dotplots were constructed in R 4.3.0 using packages ggplot2, cowplot, scales and patchwork. All other plots were constructed in Stata version 18. |

For manuscripts utilizing custom algorithms or software that are central to the research but not yet described in published literature, software must be made available to editors and reviewers. We strongly encourage code deposition in a community repository (e.g. GitHub). See the Nature Portfolio guidelines for submitting code & software for further information.

## Data

Policy information about availability of data

All manuscripts must include a data availability statement. This statement should provide the following information, where applicable:

- Accession codes, unique identifiers, or web links for publicly available datasets
- A description of any restrictions on data availability
- For clinical datasets or third party data, please ensure that the statement adheres to our policy

Data will be stored in the Swansea Trials Unit data repository and can be requested by emailing STU@swansea.ac.uk and submitting a request for access to anonymised data.

## Research involving human participants, their data, or biological material

Policy information about studies with human participants or human data. See also policy information about sex, gender (identity/presentation), and sexual orientation and race, ethnicity and racism.

| | |
|---|---|
| Reporting on sex and gender | Analysis was performed on 25 participants in the Placebo group (9 female = 36%; 16 male - 64%) and 47 participants in the Ustekinumab group (20 female = 43%; 27 male = 57%). Study design allowed recruitment of both males and females. Results apply on both sexes. The Ustekinumab and Placebo group were comparable in terms of sex. |
| Reporting on race, ethnicity, or other socially relevant groupings | Ethnicity was determined based on self-reporting. Ethnicity distribution is reported in Table 1 of the main manuscript. The Ustekinumab and Placebo group were comparable in terms of ethnicity. |
| Population characteristics | Participant included in the study were age 12-18 within 100 days of diagnosis of T1D. They had to have evidence of residual functioning beta-cells (peak serum C-peptide level >0.2nmol/L in MMTT); positive of at least one islet autoantibody (GAD, IA-2, zinc transporter protein 8 (ZnT8)); and body weight <100kg. The Ustekinumab and Placebo group were comparable in terms of sex, age, BMI, ethnicity, baseline C-peptide area under the curve (AUC) and HbA1c. |
| Recruitment | The participants were recruited from 16 paediatric and adult diabetes research centres in the United Kingdom according to inclusion and exclusion criteria defined in the Methods section of the main manuscript. Potential participants were identified from health records, clinical contacts, patient registries and self-referrals through the T1D UK consortium https://type1diabetesresearch.org.uk and ADDRESS-2 website https://www.address2.org. Wide range of recruitment sources reduced potential self-selection bias. |
| Ethics oversight | This study was carried out with the approval of the UK Research Ethics Service (approval received on 18 September 2018 from Wales Research Ethics Committee (REC) 3) reference 18/WA/0092, IRAS ID 230113, UK Medicines and Healthcare products Regulatory Agency (MHRA) for Clinical Trial Authorisation (approval received on 26 June 2018). Written informed consent or assent was obtained from all participants. The trial was conducted in compliance with the principles of the Declaration of Helsinki (2013) and the principles of Good Clinical Practice and in accordance with all applicable regulatory requirements including but not limited to the UK Policy Framework for Health and Social Care Research 2017 and the Medicines for Human Use (Clinical Trial) Regulations 2004, and subsequent amendments. |

Note that full information on the approval of the study protocol must also be provided in the manuscript.

# Field-specific reporting

Please select the one below that is the best fit for your research. If you are not sure, read the appropriate sections before making your selection.

☒ Life sciences ☐ Behavioural & social sciences ☐ Ecological, evolutionary & environmental sciences

For a reference copy of the document with all sections, see nature.com/documents/nr-reporting-summary-flat.pdf

# Life sciences study design

All studies must disclose on these points even when the disclosure is negative.

| | |
|---|---|
| Sample size | The power calculation closely followed Lachin et al.(1) based on data for children and young adolescents aged 13–17 years as well as the T1DAL study in 12–35 years (2). A sample size of 66 apportioned in a 2:1 ratio has a greater than 85% power to detect a 0.2nmol/L difference between the 2-hour MMTT mean Area Under Curve (AUC). C-peptide values of the intervention and placebo arms were assumed to be 0.5 and 0.3 (nmol/L), respectively, at 12 months. It was planned for seventy-two participants (48 ustekinumab:24 placebo) to be recruited allowing for an approximate 10% lost to follow-up.<br>1. Lachin, J.M., et al. Sample size requirements for studies of treatment effects on beta-cell function in newly diagnosed type 1 diabetes. PLoS One 6, e26471 (2011).<br>2. Rigby, M.R., et al. Targeting of memory T cells with alefacept in new-onset type 1 diabetes (T1DAL study): 12 month results of a randomised, double-blind, placebo-controlled phase 2 trial. Lancet Diabetes Endocrinol 1, 284-294 (2013). |
| Data exclusions | A total of 72 participants were randomised in a 2 : 1 ratio (in favour of treatment) and allocated to two study arms. Three eligible participants withdrew before the first treatment and were replaced. Four participants withdrew from the trial after randomisation (6%). A further four participants withdrew from treatment during the study but attended the primary endpoint assessment (week 52). In total, 68 participants attended the primary endpoint assessment (94%), of whom 64 were on treatment (89%). Six individuals were missing key baseline data required for the primary endpoint. Hence 62 participant (86%) were included in the in the Primary Outcome Measure (POM) analysis (41 in the ustekinumab group and 21 in the placebo group). |
| Replication | One sampling per participant was performed at each study point. Clinical measurements were taken at a single timepoints using GCLP measures. For the flow cytometery, assays were performed on fresh blood and hence not able to be repeated. For FLUOROSPOT, replication assays are not possible due to a limitation on the number of PBMC available. |
| Randomization | Each randomisation is via minimisation incorporating a random element and incorporates two important prognostic factors: age (12-15 versus 16-18), and screened peak C-peptide levels (0.2-0.7 vs > 0.7 nmol/L) to ensure balance between treatment groups. Sealed Envelope Ltd |

| | |
|---|---|
| | (https://sealedenvelope.com/randomisation/) will supply the minimisation algorithm and randomisation service and will host the web enabled allocation service. |
| Blinding | Double-blinded |

# Reporting for specific materials, systems and methods

We require information from authors about some types of materials, experimental systems and methods used in many studies. Here, indicate whether each material, system or method listed is relevant to your study. If you are not sure if a list item applies to your research, read the appropriate section before selecting a response.

## Materials & experimental systems

| n/a | Involved in the study | |
|---|---|---|
| ☐ | ☒ | Antibodies |
| ☒ | ☐ | Eukaryotic cell lines |
| ☒ | ☐ | Palaeontology and archaeology |
| ☒ | ☐ | Animals and other organisms |
| ☐ | ☒ | Clinical data |
| ☒ | ☐ | Dual use research of concern |
| ☒ | ☐ | Plants |

## Methods

| n/a | Involved in the study | |
|---|---|---|
| ☒ | ☐ | ChIP-seq |
| ☐ | ☒ | Flow cytometry |
| ☒ | ☐ | MRI-based neuroimaging |

## Antibodies

Antibodies used

Full details of all antibodies used are given in Supplementary Material Table 1. Dilutions are given where applicable. Details are also given below:
The following antibodies were purchased from Beckman Coulter: anti-IFN-g (Clone: 45.15; Fluorochrome: FITC; Duraclone), anti-CD8 (Clone: B9.11; Fluorochrome: PE-dazzle-594, dilution 1:50), anti-IL-4 (Clone: MP4-25D2; Fluorochrome PC7; Duraclone), anti-CD4 (Clone: 13B8.2, Fluorochrome: APC; Duraclone), anti-CD3 (Clone: UCHT1; APC-A750; Duraclone), anti-IL-17A (Clone: BL168, Fluorochrome: Pacific Blue; Duraclone), anti-CD45RA (Clone: 2H4; Fluorochrome: FITC; Duraclone), anti-CCR7 (Clone: G043H7; Fluorochrome: PE; Duraclone), anti-CD28 (Clone: CD28.2; Fluorochrome: ECD; Duraclone), anti-PD1 (Clone: PD1.3.5; Fluorochrome: PC5.5; Duraclone), anti-CD27 (Clone: A14.CD27; Fluorochrome: PC7; Duraclone), anti-CD57 (Clone: NC1; Fluorochrome: Pacific Blue; Duraclone), anti-CD45 (Clone: J33; Fluorochrome: KrOrange; Duraclone), anti-CD25 (Clone: B1.49.9; Fluorochrome: PE; Duraclone), anti-CD39 (Clone: BA54; Fluorochrome: PC5.5; Duraclone), anti-CD4 (Clone: SFCI12T4D11(T4); Fluorochrome: PC7; Duraclone), anti-FOXP3 (Clone: 259D; Fluorochrome: AF647; Duraclone), anti-Helios (Clone: 22F6; Fluorochrome: Pacific-Blue; Duraclone).

The following antibodies were purchased from Beckton Dickinson: anti-CD3 (Clone: SK7; Fluorochrome: FITC; BD Multitest™ 6-Color TBNK cocktail, dilution 1:20), anti-CD16 (Clone: B73.1; Fluorochrome: PE; BD Multitest™ 6-Color TBNK cocktail, dilution 1:20), anti-CD56 (Clone: NCAM16.2; Fluorochrome: PE; BD Multitest™ 6-Color TBNK cocktail, dilution 1:20), anti-CD45 (Clone: 2D1; Fluorochrome: PerCP-Cy5.5; BD Multitest™ 6-Color TBNK cocktail, dilution 1:20), anti-CD4 (Clone: SK3; Fluorochrome: PE-Cy7; BD Multitest™ 6-Color TBNK cocktail, dilution 1:20), anti-CD19 (Clone: SJ25C1; Fluorochrome: APC; BD Multitest™ 6-Color TBNK cocktail, dilution 1:20), anti-CD8 (Clone: SK1; Fluorochrome: APC-Cy7; BD Multitest™ 6-Color TBNK cocktail, dilution 1:20), anti-CD25 (Clone: 2A3; Fluorochrome: BV421, dilution 1:100), anti-IL-2 (Clone: MQ1-17H12; Fluorochrome: APC-R700, dilution 1:50), anti-CD25 (Clone: M-A251; Fluorochrome: PE, dilution 1:40), anti-CD56 (Clone: B159; Fluorochrome: PE-CF594, dilution 1:200), anti-CD16 (Clone: 3G8; Fluorochrome: AF700, dilution 1:200).

The following antibodies were purchased from BioLegend: anti-GM-CSF (clone: BVD2-221C11; Fluorochrome: PE-dazzle 594, dilution 1:50), anti-CD127 (Clone: A019D5; Fluorochrome: PE-dazzle 594, dilution 1;100), anti-CD25 (Clone: M-A251; Fluorochrome: BV421, dilution 1:100).

Validation

All antibodies were purchased from the vendors detailed above who performed in house target specificity and validation.

## Clinical data

Policy information about clinical studies
All manuscripts should comply with the ICMJE guidelines for publication of clinical research and a completed CONSORT checklist must be included with all submissions.

| | |
|---|---|
| Clinical trial registration | EudraCT ID 2018-000015-24; ISRCTN 14274380 |
| Study protocol | published in BMJ Open. 2021 Oct 18; 11(10):e049595. doi: 10.1136/bmjopen-2021-049595. PMID: 34663658; PMCID: PMC8524290 |
| Data collection | The trial was conducted in 16 paediatric and adult diabetes research centres in the United Kingdom: Royal London Hospital, London; Royal Alexandra Children's Hospital, Brighton; Countess of Chester Hospital, Chester; East Lancashire Hospitals NHS Trust, Burnley; The Evelina London Children's Hospital, London; Royal Devon and Exeter Hospital, Exeter; St James' Hospital, Leeds; Leicester Royal Infirmary, Leicester; Norfolk and Norwich University Hospitals, Norwich; St. George's University NHS Trust, London; University College London, London; University Hospital of Wales, Cardiff; Noah's Ark Children's Hospital, Cardiff; Swansea Bay University Health Board, |

| | |
|---|---|
| | Swansea; Ninewells Hospital, Dundee; Royal Aberdeen Children's Hospital, Aberdeen. The study was conducted from December 2018 to September 2022. |
| Outcomes | Analysis of primary outcome<br>The AUC was calculated using the trapezoidal method, not adjusted for baseline C-peptide but normalized for the 120-min period of the standard MMTT using the serum C-peptide value at each time point. Most C-peptide values fell between 0 and 1 and the distribution was positively skewed, they were transformed by log (1+x) before treatment group comparisons. These comparisons were performed with an independent T test at baseline. At Week 28 and 52, treatment group differences were assessed with ANCOVA adjusting for the baseline C-peptide value, gender, age, HbA1c and exogenous insulin use. Results were back-transformed and summarised as ratio of geometric means and percentage differences between groups49.<br>Analysis of secondary outcomes<br>Treatment group difference in secondary metabolic endpoints included HbA1c, daily insulin dose and Insulin dose-adjusted HbA1c (IDAA1c). Treatment group differences at baseline were assessed with independent T tests. Week 12, 28 and 52 treatment group differences were analysed with ANCOVA, adjusting for appropriate covariates. HbA1c and insulin use analyses post-baseline were adjusted by sex, age, HbA1c and insulin use at baseline. IDAAC was calculated according to the formula: HbA1c (%) + [4 x insulin dose (units per kg per 24 h)]64. Post-baseline IDAA1c analyses were adjusted by sex, age and IDAAC at baseline. Results were summarised as differences in arithmetic means between groups. |

## Plants

| | |
|---|---|
| Seed stocks | NA |
| Novel plant genotypes | NA |
| Authentication | NA |

## Flow Cytometry

### Plots

Confirm that:

☒ The axis labels state the marker and fluorochrome used (e.g. CD4-FITC).

☒ The axis scales are clearly visible. Include numbers along axes only for bottom left plot of group (a 'group' is an analysis of identical markers).

☒ All plots are contour plots with outliers or pseudocolor plots.

☒ A numerical value for number of cells or percentage (with statistics) is provided.

### Methodology

| | |
|---|---|
| Sample preparation | Cell surface phenotyping: 100uL of fresh EDTA blood was stained with three panels of antibodies including: (1) a modified Beckton Dickinson TBNK reagent Trucount™ tube (2) a Beckman Coulter DURAClone IM T-cell subset tube to assess maturation stages of T-cells, covering naïve, effector, memory and terminal differentiation stages and (3) a modified Beckman Coulter DURAClone Treg tube. All tubes were processed according to the manufacturer's instructions. Details of the panels are shown in Supplementary Material.<br><br>Intracellular Cytokine Staining: 100uL of fresh sodium heparin blood were stimulated with PMA-ionomycin for 3 hours using the DURActive1 DuraClone tubes (Beckman Coulter), according to the manufacturer's instructions. After the end of incubation, the blood was stained with Live Dead Yellow dye (Invitrogen) at room temperature for 20 minutes. The blood was then lysed, fixed and permeabilised using the PerFix-nc kit (Beckman Coulter), according to the manufacturer's instructions. The cells were then transferred and stained in the dark at room temperature for 45 minutes using the DuraClone IF TH tube (Beckman Coulter), with the addition of drop in antibodies targeting GM-CSF, IL-2 and CD8 PC5 as shown in Supplementary Material Table 1. The cells were then washed and acquired. |
| Instrument | Beckman Coulter Navios flow cytometer (3L 10C) |
| Software | Kaluza sofware version 2.2 (Beckman Coulter) |
| Cell population abundance | Cell population abundance was determined using BD Trucount Tubes following the manufacturers instructions but these results are not reported in this manuscript. |

Gating strategy

Gates were placed based on FMO and/or unstimulated samples

☒ Tick this box to confirm that a figure exemplifying the gating strategy is provided in the Supplementary Information.
